# Supplementary material for: Structures of influenza A and B replication complexes give insight into avian to human host adaptation and reveal a role of ANP32 as an electrostatic chaperone for the apo-polymerase
Source: Nat Commun. 2024 Aug 19;15:6910. doi: 10.1038/s41467-024-51007-3 (PMC11333492; doi:10.1038/s41467-024-51007-3)
Supplement: Supplementary file 2 — Supplementary Information [file 41467_2024_51007_MOESM2_ESM.pdf]

Supplemental information

Supplementary Table 1-4. Cryo-EM data collection, refinement and validation statistics.

Supplementary Figures 1-11.

Supplementary Notes 1-7, 9. Cryo-EM data processing and map quality.

Supplementary Notes 8. ANP32 multiple sequence alignment.

Supplementary References

Supplementary Table 1. Cryo-EM data collection, refinement and validation statistics for FluPol A/H7N9-4M with hANP32A.

Sample	FluPol A/H7N9-4M with hANP32A				
Structure	Endo(R) core1	Endo(R) core2	Focus replicase minus 627(R)	Focus Encapsidase plus 627(R) hANP32A	Complete replication complex
PDB ID	PDB ID 8RMP	PDB ID 8RMQ	PDB ID 8RMS	PDB ID 8RN0	PDB ID 8RMR
EMDB ID	EMD-19366	EMD-19367	EMD-19369	EMD-19382	EMD-19368
**Data collection and processing**					
Microscope	ThermoFisher Krios TEM				
Voltage (kV)	300				
Camera	Gatan K3 direct electron detector mounted on a Gatan Bioquantum LS/967 energy filter				
Magnification	105,000				
Nominal defocus range (µm)	-0.8 / -2				
Electron exposure (e–/Å^2)	40				
Pixel size (Å)	0.84				
Initial micrographs (no.)	14,001				
Final micrographs (no.)	13,328				
**Refinement**					
Particles per class (no.)	56,572	277,339	18,596		
Map resolution (Å), 0.143 FSC	2.77	2.54	3.21	3.13	3.25
Model resolution (Å), 0.5 FSC	3.0	2.6	3.76	3.60	3.80
Map sharpening *B* factor (Å^2)	-75	-87	-56	-56	-55
Map versus model cross-correlation (CCmask)	0.8241	0.8462	0.7118	0.8241	0.8462
**Model composition**					
Non-hydrogen atoms	13818	13676	16190	18082	34215
Protein residues	1717	1699	2019	2262	4273
Water	-	7	-	-	-
Ligands	1 Mg	2 Mg	1 Mg	-	2 Mg
***B* factors (Å^2)**					
Protein	79.96	49.91	99.99	116.09	100.81
Water	-	21.56	-	-	-
**RMS deviations**					
Bond lengths (Å)	0.003	0.003	0.002	0.002	0.002

Bond angles (°)	0.512	0.484	0.466	0.482	0.502
**Validation**					
MolProbity score	1.76	1.65	1.58	1.76	1.77
All-atom clash score	4.49	4.57	7.06	8.05	8.85
Poor rotamers (%)	2.95	2.45	0.17	0.15	0.26
**Ramachandran plot**					
Favored (%)	97.00	97.38	96.85	95.44	95.73
Allowed (%)	2.94	2.62	3.15	4.43	4.20
Outliers (%)	0.06	0.0	0.0	0.13	0.07

Supplementary Table 2. Cryo-EM data collection, refinement and validation statistics for FluPol B/Memphis with hANP32A.

Sample	FluPol B/Memphis with hANP32A				
**Structure**	**Focus replicase minus 627(R)**	**Focus Encapsidase plus 627(R) and hANP32A**	**Complete replication complex**	**Complete Trimer**	**Monomer**
PDB ID	PDB ID 8RN9	PDB ID 8RNB	PDB ID 8RNC	PDB ID 8RNA	PDB ID 8RN2
EMDB ID	EMD-19391	EMD-19393	EMD-19394	EMD-19392	EMD-19384
**Data collection and processing**					
Microscope	ThermoFisher Krios TEM				
Voltage (kV)	300				
Camera	Gatan K3 direct electron detector mounted on a Gatan Bioquantum LS/967 energy filter				
Magnification	105,000				
Nominal defocus range (μm)	-0.8 / -2.0				
Electron exposure (e–/Å^2)	40				
Pixel size (Å)	0.84				
Initial micrographs (no.)	15,650				
Final micrographs (no.)	15,234				
**Refinement**					
Particles per class (no.)	24,051				167,036
Map resolution (Å), 0.143 FSC	3.31	3.13	3.52	3.57	2.89
Model resolution (Å), 0.5 FSC	3.6	3.6	Composite model not refined	Composite model not refined	3.1
Map sharpening *B* factor (Å^2)	-59	-61	-64	-61	-98
Map versus model cross-correlation (CCmask)	0.7848	0.7892	Composite model not refined	Composite model not refined	0.7558
**Model composition**					
Non-hydrogen atoms	17876	20714	35638	45474	15504
Protein residues	2246	2594	4477	5718	1952
**Mean *B* factor (Å^2)**					
Protein	87.56	110.96	93.41	88.31	70.96
**RMS deviations**					

Bond lengths (Å)	0.003	0.002	0.002	0.002	0.003
Bond angles (°)	0.529	0.487	0.508	0.505	0.543
**Validation**					
MolProbity score	1.77	1.76	1.80	1.74	1.95
All-atom clash score	7.94	7.94	8.60	8.00	6.98
Poor rotamers (%)	0.31	0.40	0.33	0.68	2.11
**Ramachandran plot**					
Favored (%)	95.15	95.25	95.22	95.64	95.46
Allowed (%)	4.81	4.67	4.71	4.29	4.54
Outliers (%)	0.04	0.08	0.07	0.07	0.0

Supplementary Table 3. Cryo-EM data collection, refinement and validation statistics for FluPol B/Memphis pseudo-symmetric dimer.

Sample	FluPol B/Memphis with hANP32A					
**Structure**	**Symmetric dimer focus encapsidase**	**Symmetric dimer Encap+ Focus Endo(R)**	**Symmetric dimer Encap+ Focus Endo(E)**	**Symmetric dimer Encap+ Focus Endo(T)**	**Symmetric dimer Encap+ Focus Core**	**Complete Sym dimer Encap+Mix**
PDB ID	PDB ID 8RN3	PDB ID 8RN5	PDB ID 8RN6	PDB ID 8RN4	PDB ID 8RN7	PDB ID 8RN8
EMDB ID	EMD-19385	EMD-19387	EMD-19388	EMD-19386	EMD-19389	EMD-19390
**Data collection and processing**						
Microscope	ThermoFisher Krios TEM					
Voltage (kV)	300					
Camera	Gatan K3 direct electron detector mounted on a Gatan Bioquantum LS/967 energy filter					
Magnification	105,000					
Nominal defocus range (µm)	-0.8 / -2.0					
Electron exposure (e–/Å^2)	40					
Pixel size (Å)	0.84					
Initial micrographs (no.)	15,650					
Final micrographs (no.)	15,234					
**Refinement**						
Particles per class (no.)	179,550	92,684	88,053	86,279	34,404	179,550
Map resolution (Å), 0.143 FSC	2.75	2.88 (dimer 3.07)	2.82 (dimer 3.0)	2.87 (dimer 3.10)	3.09 (dimer 3.38)	2.92
Model resolution (Å), 0.5 FSC	2.9	3.1	3.0	3.1	3.3	3.0
Map sharpening *B* factor (Å^2)	-90	-93	-94	-94	-79	-100
Map versus model cross-correlation (CCmask)	0.8543	0.7680	0.8671	0.8160	0.7917	0.8087
**Model composition**						
Non-hydrogen atoms	15528	16487	11544	13718	12105	25662
Protein residues	1955	2074	1455	1721	1524	3234
Water	-	-	-	-	-	-

Ligands	2 Mg	1 Mg	-	2 Mg	1 Mg	5 Mg
**Mean *B* factors (Å^2)**						
Protein	39.07	107.66	55.67	50.32	83.82	59.20
Water	-					-
**RMS deviations**						
Bond lengths (Å)	0.003	0.002	0.003	0.002	0.002	0.003
Bond angles (°)	0.574	0.473	0.551	0.478	0.483	0.495
**Validation**						
MolProbity score	2.11	1.91	1.88	1.63	1.63	1.81
All-atom clash score	7.65	6.13	5.85	5.13	6.96	6.27
Poor rotamers (%)	3.05	2.54	2.21	1.73	0.0	1.95
**Ramachandran plot**						
Favored (%)	95.31	96.15	95.69	97.00	96.34	96.35
Allowed (%)	4.53	3.80	4.24	2.94	3.60	3.56
Outliers (%)	0.15	0.05	0.07	0.06	0.07	0.09

Supplementary Table 4. Cryo-EM data collection, refinement and validation statistics for FluPol B/Memphis encapsidase bound to 5' cRNA 1-12.

**Sample**	**FluPol B/Memphis with 5' cRNA 1-12**
**Structure**	
PDB ID	PDB ID 8RN1
EMDB ID	EMD-19383
**Data collection and processing**	
Microscope	Glacios
Voltage (kV)	200
Camera	Falcon 4i / SelectrisX
Magnification	130,000
Nominal defocus range (µm)	-0.8 / -2.0
Electron exposure (e–/Å^2)	40
Pixel size (Å)	0.878
Initial micrographs (no.)	2,451
Final micrographs (no.)	2,353
**Refinement**	
Particles per class (no.)	15,009
Map resolution (Å), 0.143 FSC	3.64
Model resolution (Å), 0.5 FSC	3.85
Map sharpening *B* factor (Å^2)	-90
Map versus model cross-correlation (CCmask)	0.8013
**Model composition**	
Non-hydrogen atoms	16468
Protein residues	2037
RNA nts	12
**Mean *B* factors (Å^2)**	
Protein	109.41
RNA	75.42
**RMS deviations**	
Bond lengths (Å)	0.003
Bond angles (°)	0.532
**Validation**	
MolProbity score	1.84
All-atom clash score	10.27
Poor rotamers (%)	0.34
**Ramachandran plot**	
Favored (%)	95.50
Allowed (%)	4.50
Outliers (%)	0.0

SUPPLEMENTARY FIGURE 1

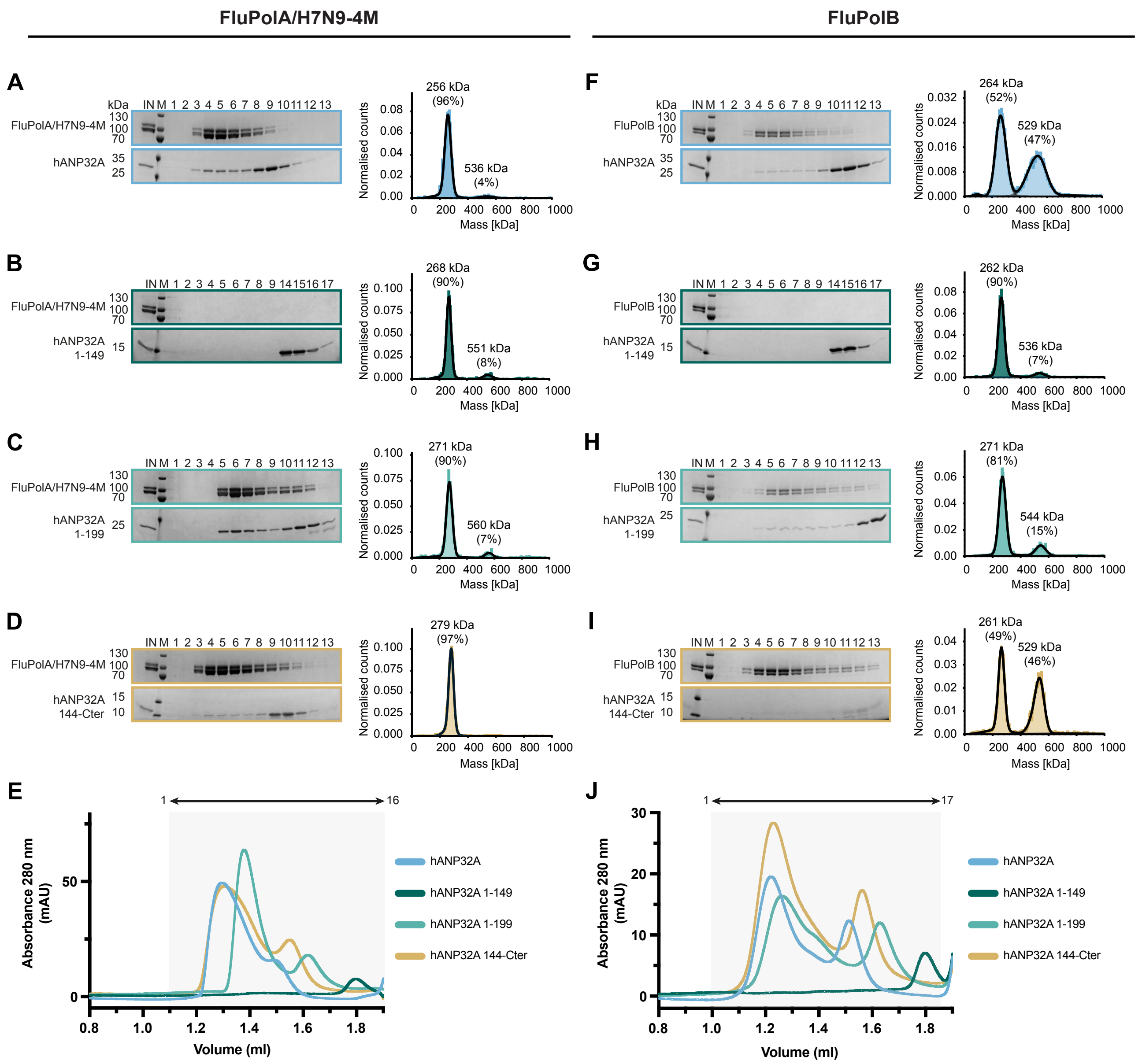

Supplementary Figure 1. Biochemical analysis of the interaction of FluPolA/H7N9-4M and FluPolB with different hANP32A constructs.

(A) SDS-PAGE and mass photometry analysis of FluPolA/H7N9-4M-hANP32A at 150 mM NaCl. The molecular ladder (M) in kDa, FluPolA/H7N9-4M heterotrimer and hANP32A are indicated on the left of the gel. “IN” corresponds to the input. This data is also presented in Figure 1F.

(B) SDS-PAGE and mass photometry analysis of FluPolA/H7N9-4M interaction with hANP32A 1-149 (LRR domain alone) at 150 mM NaCl.

(C) SDS-PAGE and mass photometry analysis of FluPolA/H7N9-4M interaction with hANP32A 1-199 (LRR domain with half the LCAR) at 150 mM NaCl.

(D) SDS-PAGE and mass photometry analysis of FluPolA/H7N9-4M interaction with hANP32A 144-C-terminus (LCAR alone) at 150 mM NaCl.

(E) Superposition of size exclusion chromatography (SEC) profiles of FluPolA/H7N9-4M with hANP32A (blue), hANP32A 1-149 (dark green), hANP32A 1-199 (green) and hANP32A 144-C-terminus (yellow) at 150 mM NaCl. SEC profile of FluPolA/H7N9-4M with hANP32A is presented in Figure 1H. The relative absorbance at 280 nm (mAU) is on the y-axis. The elution volume (ml) is on the x-axis, graduated every 50 µl. SDS-PAGE fractions 1 to 17 corresponds to the elution volume 1.1 ml - 1.95 ml, represented as an arrow on top.

(F) SDS-PAGE and mass photometry analysis of FluPolB-hANP32A at 150 mM NaCl. The molecular ladder (M) in kDa, FluPolB heterotrimer and hANP32A are indicated on the left of the gel. “IN” corresponds to the input. This data is also presented in Figure 1N.

(G) SDS-PAGE and mass photometry analysis of FluPolB interaction with hANP32A 1-149 (LRR domain alone) at 150 mM NaCl.

(H) SDS-PAGE and mass photometry analysis of FluPolB interaction with hANP32A 1-199 (LRR domain with half the LCAR) at 150 mM NaCl.

(I) SDS-PAGE and mass photometry analysis of FluPolB interaction with hANP32A 144-C-terminus (LCAR alone) at 150 mM NaCl.

(J) Superposition of SEC profiles of FluPolB with hANP32A (blue), hANP32A 1-149 (dark green), hANP32A 1-199 (green) and hANP32A 144-C-terminus (yellow) at 150 mM NaCl. SEC profile of FluPolB with hANP32A is presented in Figure 1P. The relative absorbance at 280 nm (mAU) is on the y-axis. The elution volume (ml) is on the x-axis, graduated every 50 µl. SDS-PAGE fractions 1 to 17 corresponds to the elution volume 1.0 ml - 1.85 ml, represented as an arrow on top.

Source data are provided as a Source Data file (n=1-3 independent experiments).

SUPPLEMENTARY FIGURE 2

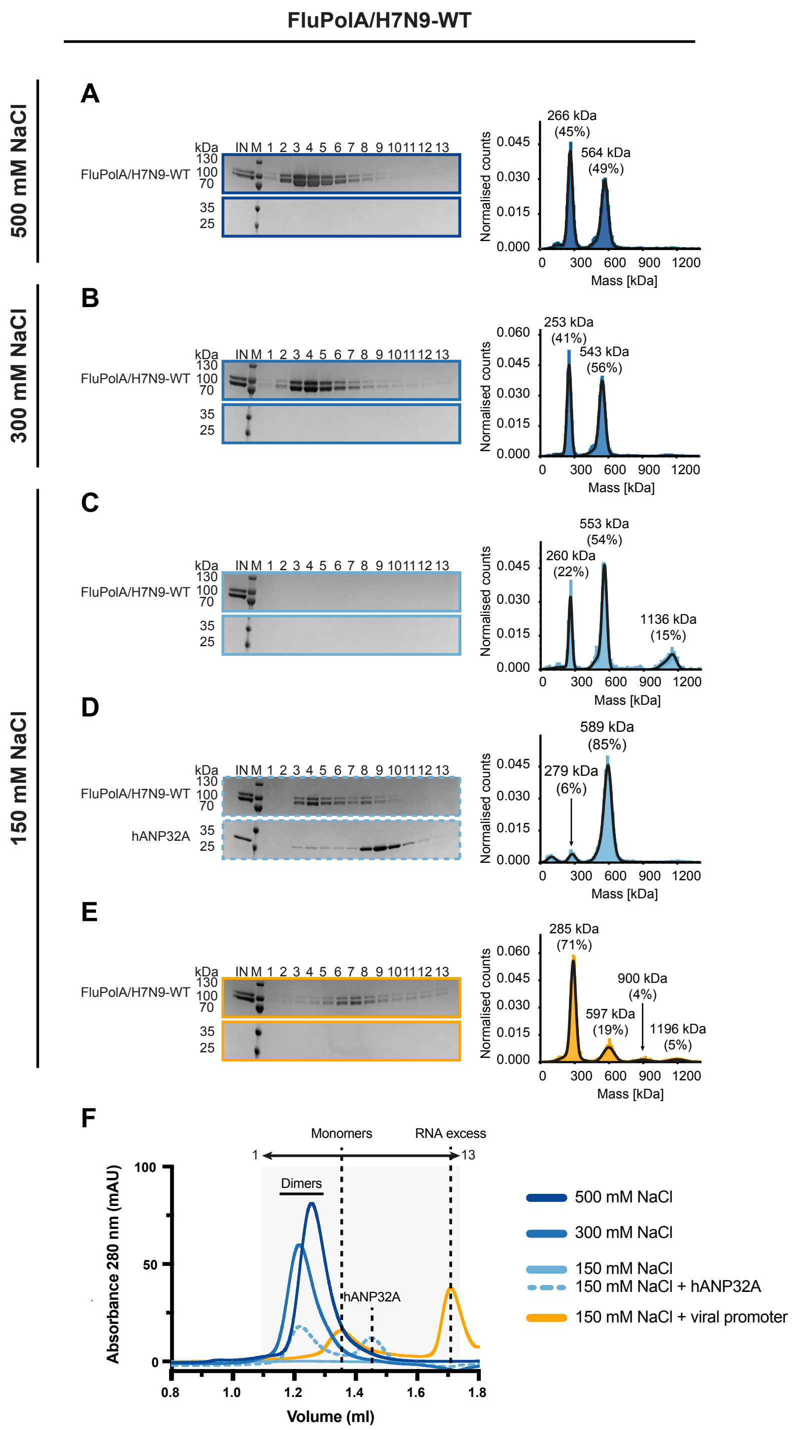

Supplementary Figure 2. Biochemical analysis of the interaction of FluPolA/H7N9-WT with hANP32A and viral promoter.

(A-C) SDS-PAGE and mass photometry analysis of FluPolA/H7N9-WT at (A) 500 mM NaCl, (B) 300 mM and (C) 150 mM NaCl without hANP32A. The molecular ladder (M) in kDa, FluPolA/H7N9-WT heterotrimer are indicated on the left of the gel. “IN” corresponds to the input.

(D) SDS-PAGE and mass photometry analysis of FluPolA/H7N9-WT interaction with hANP32A at 150 mM NaCl.

(E) SDS-PAGE and mass photometry analysis of FluPolA/H7N9-WT in complex with vRNA promoter bound.

(F) Superposition of size exclusion chromatography profiles of FluPolA/H7N9-WT at 500 mM NaCl (dark blue), 300 mM NaCl (medium blue), 150 mM NaCl (light blue), 150 mM NaCl with hANP32A (dotted line, light blue), 150 mM NaCl with vRNA promoter bound (orange). The relative absorbance at 280 nm (mAU) is on the y-axis. The elution volume (ml) is on the x-axis, graduated every 50 µl. SDS-PAGE fractions 1 to 13 corresponds to the elution volume 1.1 ml - 1.75 ml, represented as an arrow on top.

Source data are provided as a Source Data file (n=1-3 independent experiments).

SUPPLEMENTARY FIGURE 3

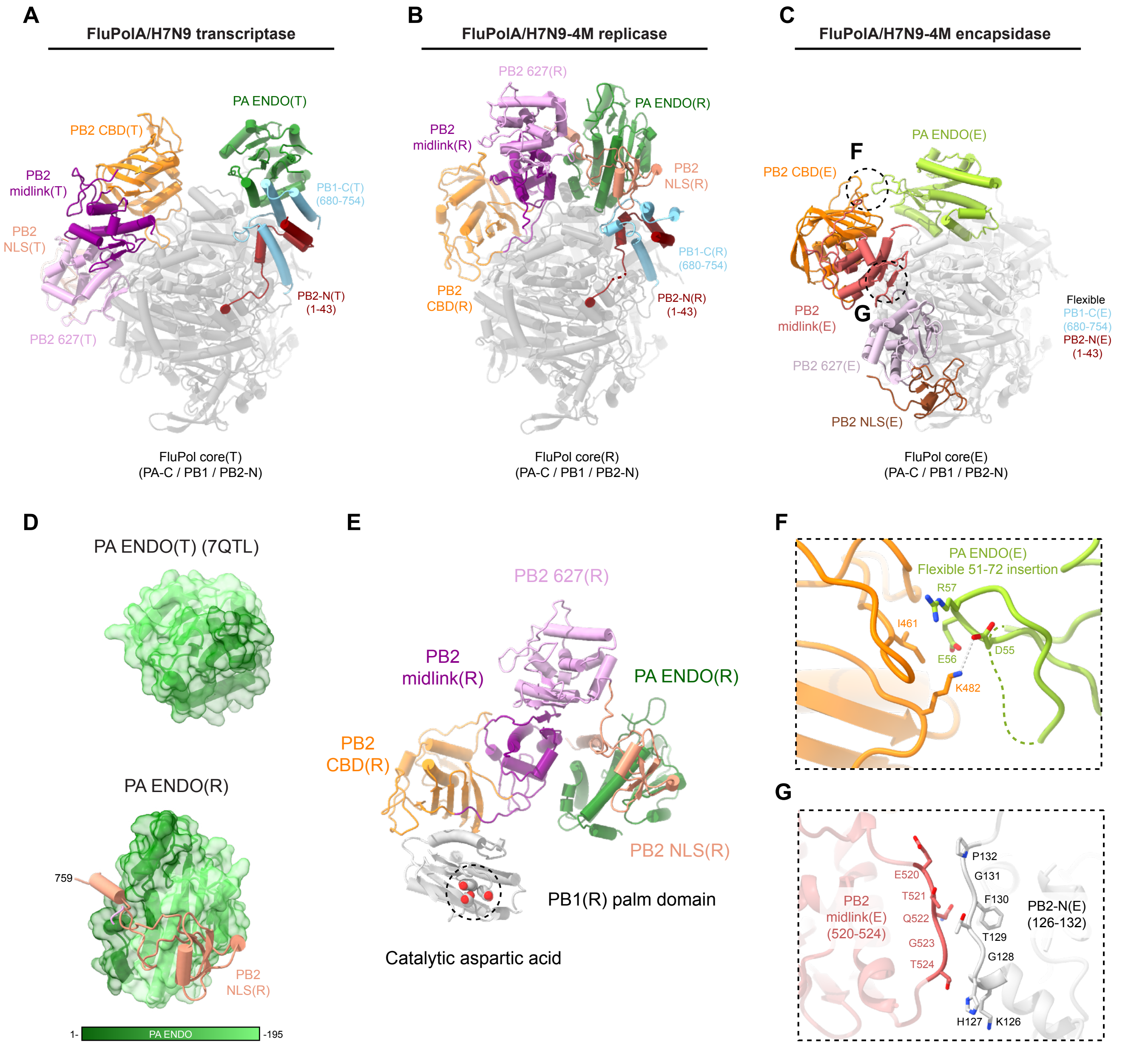

Supplementary Figure 3. Structural comparison of FluPolA/H7N9 transcriptase, replicase and encapsidase conformations.

(A) Cartoon representation of FluPolA/H7N9 in the transcriptase conformation (FluPolA/H7N9(T)) (PDB 7QTL). FluPolA/H7N9(T) core is dark grey, shown in transparency, PA ENDO(T) in dark green, PB1-C(T) in blue, PB2-N(T) in red, PB2 midlink(T) in magenta, PB2 CBD(T) in orange, PB2 627(T) in pink, PB2 NLS(T) in beige.

(B) Cartoon representation of FluPolA/H7N9-4M in the replicase conformation (FluPolA/H7N9-4M(R)), extracted from the replication complex and aligned on FluPolA/H7N9(T) PB1 subunit. FluPolA/H7N9-4M(R) core is in dark grey, shown in transparency, PA ENDO(R) in dark green, PB1-C(R) in blue, PB2-N(R) in red, PB2 midlink(R) in magenta, PB2 CBD(R) in orange, PB2 627(R) in pink, PB2 NLS(R) in beige.

(C) Cartoon representation of FluPolA/H7N9-4M in the encapsidase conformation (FluPolA/H7N9-4M(E)), extracted from the replication complex and aligned on FluPolA/H7N9(T) PB1 subunit. FluPolA/H7N9-4M(E) core is in light grey, shown in transparency, PA ENDO(E) in light green, PB2 midlink(E) in salmon, PB2 CBD(E) in orange, PB2 627(E) in light pink, PB2 NLS(E) in brown. PB1-C(E) and PB2-N(E) are flexible. Specific interactions within FluPolA/H7N9-4M(E) are annotated with dotted black circles, and refer to panels (F) and (G).

(D) Comparison of the PA-endonuclease (ENDO) conformation between FluPolA/H7N9(T) and FluPolA/H7N9-4M(R). ENDOs are displayed as transparent surface, coloured from the N-terminus to the C-terminus from dark to light green. ENDO(R) rotates and interacts with PB2 NLS(R), represented as cartoon and coloured in beige.

(E) Cartoon representation of PB2(R) C-terminal domains and PB1(R) palm domain. PB2(R) C-terminal domains are coloured as in (B). PB2 CBD(R) interacts with PB1 palm domain, in light grey. Catalytic aspartic acids are shown with atoms as spheres, circled with a dotted line.

(F) Close-up view of the flexible insertion (51-72) of PA ENDO(E) interacting with PB2 CBD(E). Domains are coloured as in (C). PA ENDO(E) residues 67-72 are flexible and represented as a dotted line. Ionic and hydrogen bonds are shown as grey dotted lines.

(G) Close-up view of the interaction between PB2-N(E) and PB2 midlink(E). Domains are coloured as in (C). Interacting residues are displayed.

SUPPLEMENTARY FIGURE 4

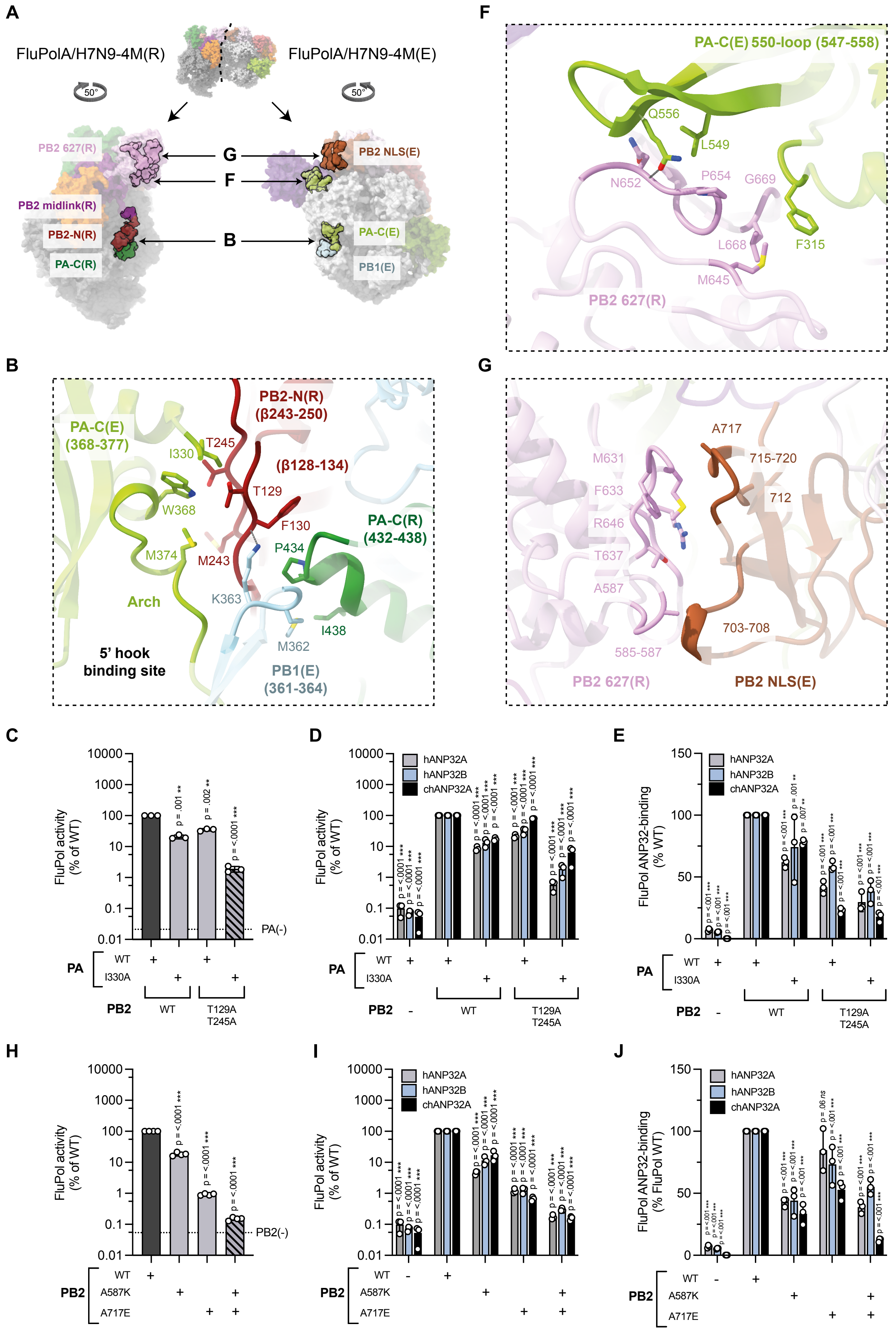

Supplementary Figure 4. Interface between FluPolA/H7N9-4M(R) and FluPolA/H7N9-4M(E).

(A) Overview of the interacting domains between FluPolA/H7N9-4M(R) and FluPolA/H7N9-4M(E). Both replicase and encapsidase moiety were split and rotated by 50 degrees. Interacting surface is shown as non-transparent. Most domains are coloured as in Figure 2, with PA(R) in dark green, PB2-N(R) in red. For the three main interfaces, a close-up view is shown in panels (B), (F), (G).

(B) FluPolA/H7N9-4M PA-C(R), PB2-N(R) and FluPolA/H7N9-4M PA(E) arch interaction. Domains are coloured as in (A). Interacting residues are displayed, shown as non-transparent. Ionic and hydrogen bonds are shown as grey dotted lines.

(C) Mutational analysis of the PB2-N(R) - PA(E) arch interaction shown in (B). Cell-based assay of WSN FluPol activity for the indicated PA and PB2 mutants and combinations thereof. HEK-293T cells were co-transfected with plasmids encoding PB2, PB1, PA, NP with a model vRNA encoding the Firefly luciferase. Luminescence was normalised to a transfection control and is represented as a percentage of FluPol WT (mean   SD, n=3, **p < 0.002, ***p < 0.001, one-way ANOVA; Dunnett's multiple comparisons test). Source data are provided as a Source Data file.

(D) Mutational analysis of the PB2-N(R) - PA(E) arch interaction shown in (B). Cell-based assay of WSN FluPol activity for the indicated PA and PB2 mutants and combinations thereof. HEK-293T in which hANP32A and hANP32B were knocked out were transfected as in (C) and transiently complemented by co-transfection of plasmids encoding hANP32A, hANP32B or chANP32A. Luminescence was normalised to a transfection control and is represented as a percentage of FluPol WT (mean   SD, n=3, ***p < 0.001, two-way ANOVA; Dunnett's multiple comparisons test). Source data are provided as a Source Data file.

(E) Mutational analysis of the PB2-N(R) - PA(E) arch interaction as shown in (B). Cell-based assay of WSN FluPol binding to ANP32 for the indicated PA and PB2 mutants and combinations thereof. HEK-293T cells were co-transfected with plasmids encoding PB2, PA, PB1-luc1 and either hANP32A-luc2, hANP32B-luc2 or chANP32A-luc2. Luminescence signals due to luciferase reconstitution are represented as a percentage of FluPol WT (mean   SD, n=3, **p < 0.002, ***p < 0.001, two-way ANOVA; Dunnett's multiple comparisons test). Source data are provided as a Source Data file.

(F) FluPolA/H7N9-4M PB2 627(R) C-terminal β-sheet and FluPolA/H7N9-4M PA-C 550-loop(E) interaction. Domains are coloured as in (A). Interacting residues are displayed, shown as non-transparent. Ionic and hydrogen bonds are shown as grey dotted lines.

(G) FluPolA/H7N9-4M PB2 627(R) and FluPolA/H7N9-4M PB2 NLS(E) interaction. Domains are coloured as in (A). Most of interacting residues are displayed, shown as non-transparent.

(H) Mutational analysis of the PB2-627(R) - PB2-NLS(E) interaction as shown in (G). Cell-based assay of WSN FluPol activity for the indicated PB2 mutants and combinations thereof as described in (C) (mean   SD, n=4, ***p < 0.001, one-way ANOVA; Dunnett's multiple comparisons test). Source data are provided as a Source Data file.

(I) Mutational analysis of the PB2-627(R) - PB2-NLS(E) interaction as shown in (G). Cell-based assay of WSN FluPol activity for the indicated PB2 mutants and combinations thereof as described in (D) (mean   SD, n=3, ***p < 0.001, two-way ANOVA; Dunnett's multiple comparisons test). Source data are provided as a Source Data file.

(J) Mutational analysis of the PB2-627(R) - PB2-NLS(E) interaction as shown in (G). Cell-based assay of WSN FluPol binding to ANP32 for the indicated PB2 mutants and combinations thereof as described in (E) (mean   SD, n=3, ***p < 0.001, two-way ANOVA; Dunnett's multiple comparisons test). Source data are provided as a Source Data file.

SUPPLEMENTARY FIGURE 5

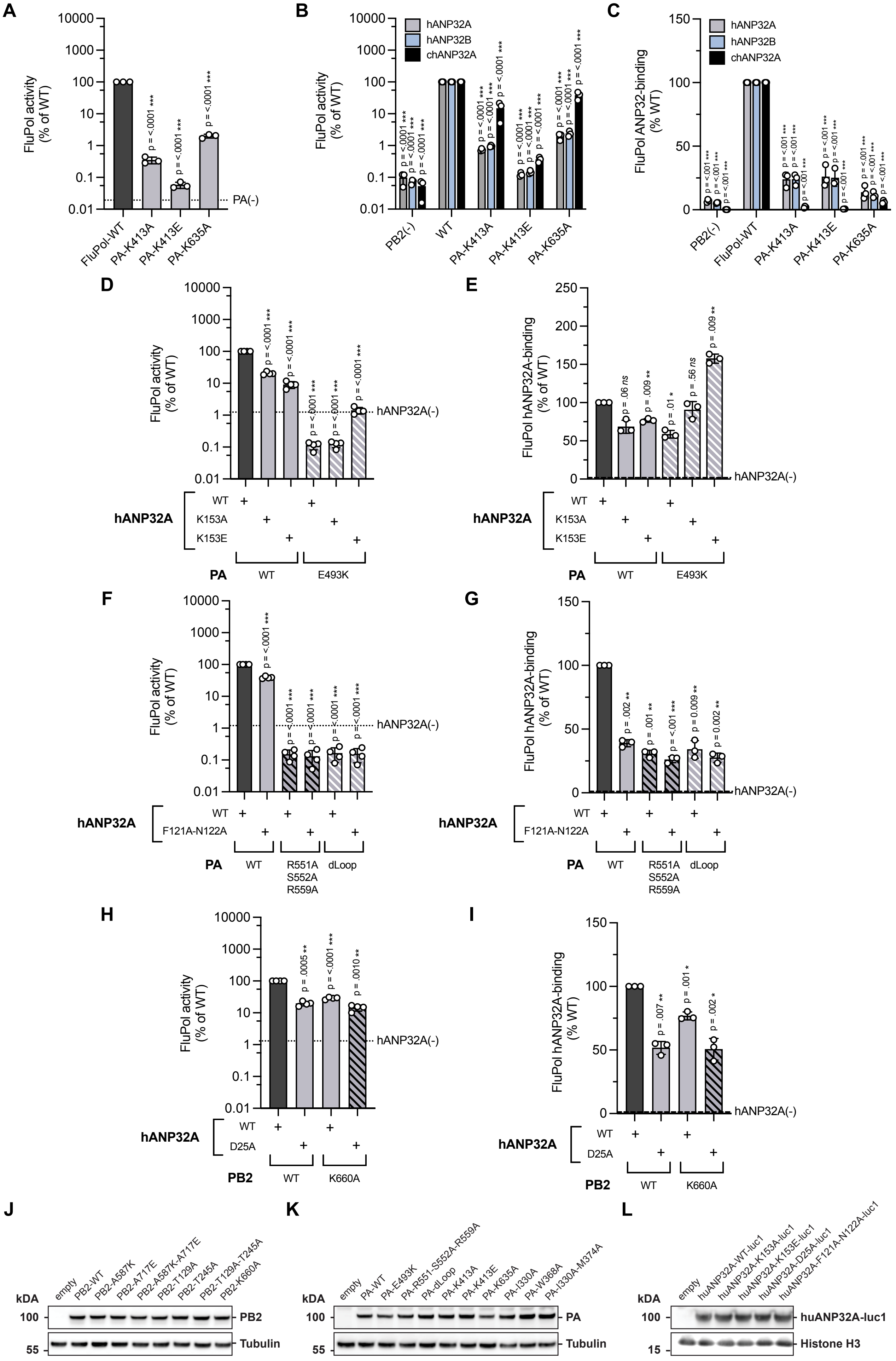

Supplementary Figure 5. Interface between FluPolA/H7N9-4M and hANP32A.

(A) Mutational analysis of the PA(E)-hANP32A interaction shown in Fig. 2C. Cell-based assay of WSN FluPol activity for the indicated PA mutants. HEK-293T WT cells were co-transfected with plasmids encoding PB2, PB1, PA, NP with a model vRNA encoding the Firefly luciferase. Luminescence was normalised to a transfection control and is represented as a percentage of PA-WT (mean SD, n=3, ***p < 0.001, one-way ANOVA; Dunnett's multiple comparisons test).

(B) Mutational analysis of the PA(E)-hANP32A interaction shown in Fig. 2C. Cell-based assay of WSN FluPol activity for the indicated PA mutants. HEK-293T in which hANP32A and hANP32B were knocked out were transfected as in (A) and transiently complemented by co-transfection of plasmids encoding hANP32A, hANP32B or chANP32A. Luminescence was normalised to a transfection control and is represented as a percentage of FluPol WT (mean SD, n=3, ***p < 0.001, two-way ANOVA; Dunnett's multiple comparisons test).

(C) Mutational analysis of the PA(E)-hANP32A interaction shown in Fig. 2C. Cell-based assay of WSN FluPol binding to ANP32 for the indicated PA mutants. HEK-293T cells were co-transfected with plasmids encoding PB2, PA, PB1-luc1 and either hANP32A-luc2, hANP32B-luc2 or chANP32A-luc2. Luminescence signals due to luciferase reconstitution are represented as a percentage of FluPol WT (mean SD, n=3, ***p < 0.001, two-way ANOVA; Dunnett's multiple comparisons test).

(D) Mutational analysis of the PA(E)-hANP32A interaction shown in Fig. 2D. Cell-based assay of WSN FluPol activity for the indicated PA and hANP32A mutants. HEK-293T in which hANP32A and hANP32B were knocked out were transfected as in (A) and transiently complemented by co-transfection of plasmids encoding hANP32A-WT or the indicated hANP32A mutants. Luminescence was normalised to a transfection control and is represented as a percentage of WT (mean SD, n=4, ***p < 0.001, one-way ANOVA; Dunnett's multiple comparisons test).

(E) Mutational analysis of the PA(E)-hANP32A interaction shown in Fig. 2D. Cell-based assay or WSN FluPol binding to ANP32 for the indicated PA and hANP32A mutants as described in (C) (mean SD, n=3, *< 0.033, **p < 0.002, one-way ANOVA; Dunnett's multiple comparisons test).

(F) Mutational analysis of the PA(E)-hANP32A interaction shown in Fig. 2E. Cell-based assay of WSN FluPol activity for the indicated PA and hANP32A mutants as described in (D) (mean SD, n=4, ***p < 0.001, one-way ANOVA; Dunnett's multiple comparisons test).

(G) Mutational analysis of the PA(E)-hANP32A interaction shown in Fig. 2E. Cell-based assay of WSN FluPol binding to ANP32 for the indicated PA and hANP32A mutants as described in (C) (mean SD, n=3, **p < 0.002, ***p < 0.001, one-way ANOVA; Dunnett's multiple comparisons test).

(H) Mutational analysis of the PB2(R)-hANP32A interaction shown in Fig. 2F. Cell-based assay of WSN FluPol activity for the indicated PB2 and hANP32A mutants as described in (D) (mean SD, n=4, **p < 0.002, ***p < 0.001, one-way ANOVA; Dunnett's multiple comparisons test).

(I) Mutational analysis of the PB2(R)-hANP32A interaction shown in Fig. 2F. Cell-based WSN FluPol ANP32-binding assays of the indicated PB2 and hANP32A mutants as described in (C) (mean SD, n=3, *< 0.033, **p < 0.002, one-way ANOVA; Dunnett's multiple comparisons test).

(J-K) HEK-293T cells were co-transfected with expression plasmids for WSN PB1, PB2 and PA with the indicated PB2 (J) or PA (K) mutations. Cell lysates were analysed by western blot using antibodies specific for PB2, PA and tubulin. Uncropped gels are provided as a source data file.

(L) HEK-293T cells were transfected with expression plasmids for hANP32A-luc1 with the indicated mutations. Cell lysates were analysed by western blot using antibodies specific for Gaussia luciferase and Histone H3. Uncropped gels are provided as a source data file.

Source data are provided as a Source Data file.

SUPPLEMENTARY FIGURE 6

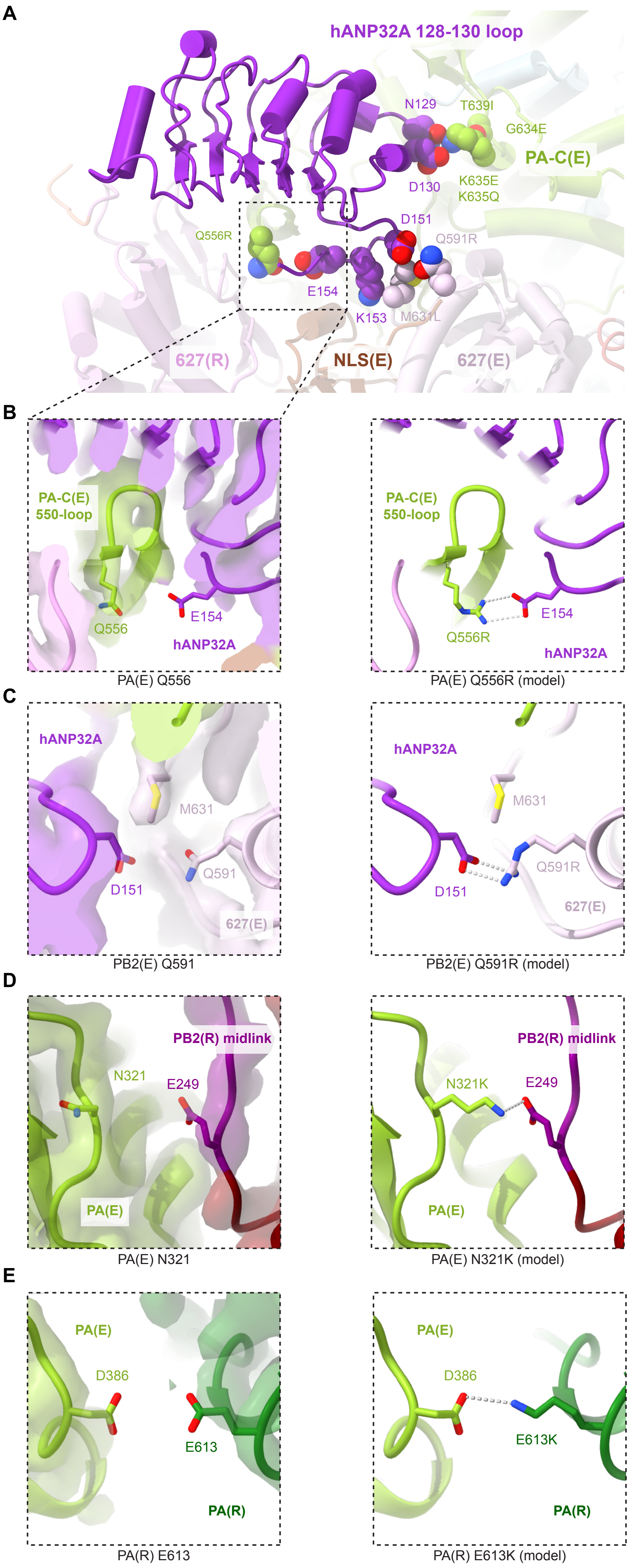

Supplementary Figure 6. Human adaptive mutations mapped onto the FluPolA/H7N9-4M replication complex.

(A) Overview of hANP32A interaction with FluPolA/H7N9-4M(R) and FluPolA/H7N9-4M(E). Domains are coloured as in Figure 2. FluPolA/H7N9-4M adaptive mutations are annotated (PA Q556R, G634E K635E/Q, T639I and PB2 Q591R, M631L). Corresponding residues of the FluPolA/H7N9-4M replication complex structure are displayed. Atoms are shown as spheres.

(B) Close-up view of PA-C(E) showing the effect of the Q556R mutation. Left: PA-C(E) Q556 residue as built in the FluPolA/H7N9-4M replication complex structure. Right: Modelled PA-C(E) Q556R mutation is likely to make a salt-bridge with hANP32A E154. Ionic bonds are shown as grey dotted lines. Coulomb potential map is shown.

(C) Close-up view of PB2(E) showing the effect of the Q591R mutation. Left: PB2(E) Q591 residue as built in the FluPolA/H7N9-4M replication complex structure. Right: Modelled PB2(E) Q591R mutation is likely to make a salt-bridge with hANP32A D151. Ionic bonds are shown as grey dotted lines. Coulomb potential map is shown.

(D) Close-up view of PA(E) showing the effect of the N321K mutation. Left: PA(E) N321 residue as built in the FluPolA/H7N9-4M replication complex structure. Right: Modelled PA(E) N321K mutation is likely to reinforce the replicase-encapsidase interface, by interacting with PB2 midlink(R) E249. Ionic bonds are shown as grey dotted lines. Coulomb potential map is shown.

(E) Close-up view of PA(R) showing the effect of the E613K mutation. Left: PA(R) E613 residue as built in the FluPolA/H7N9-4M replication complex structure. Right: Modelled PA(R) E613K mutation is likely to reinforce the replicase-encapsidase interface, by interacting with PA(E) D386. Ionic bonds are shown as grey dotted lines. Coulomb potential map is shown.

SUPPLEMENTARY FIGURE 7

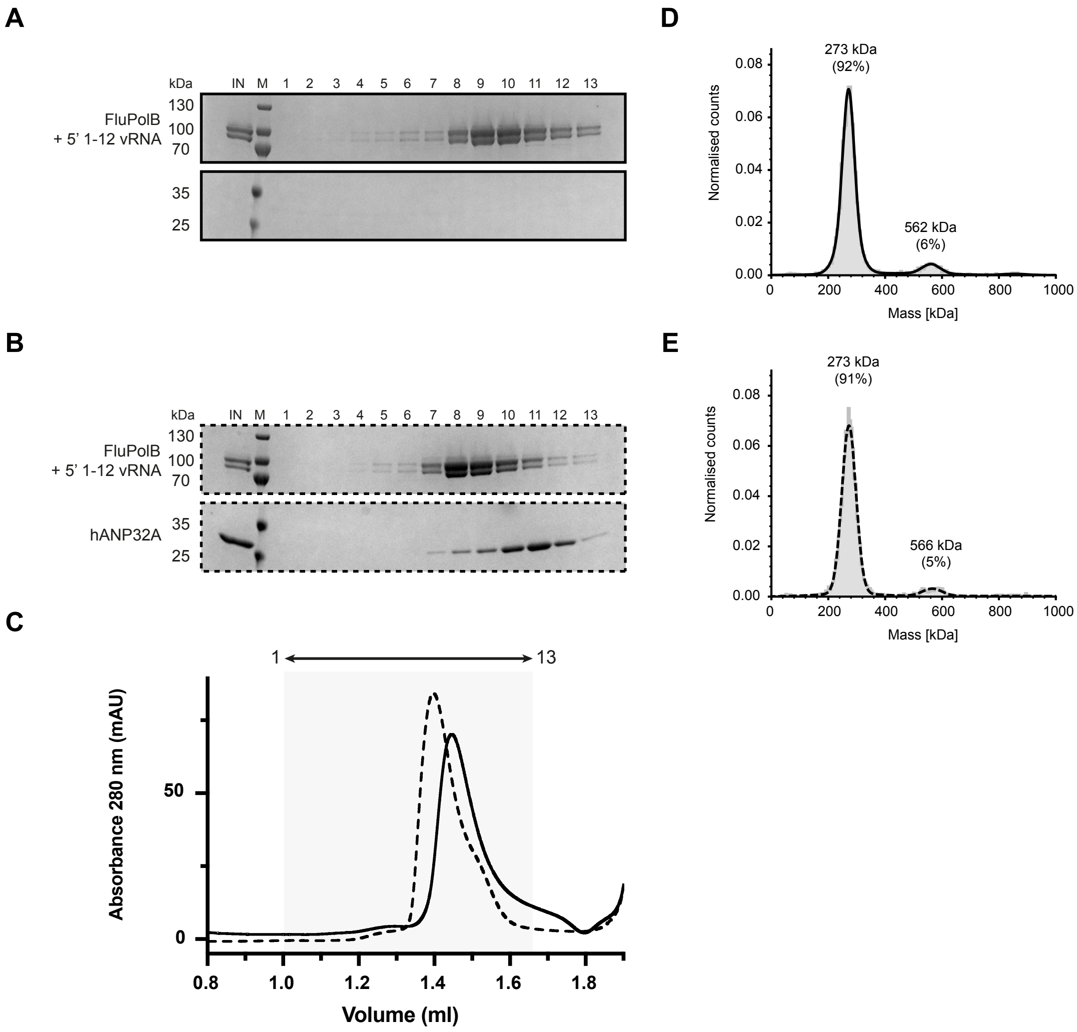

Supplementary Figure 7. 5′ vRNA end binding dissociates FluPolB dimer.

(A) SDS-PAGE analysis of FluPolB bound to the 5′ vRNA end (nucleotides 1-12) at 150 mM NaCl. The molecular ladder (M) in kDa and FluPolB heterotrimer are indicated on the left of the gel. "IN" corresponds to the input.

(B) SDS-PAGE analysis of FluPolB bound to the 5′ vRNA end (1-12) with excess of hANP32A at 150 mM NaCl. The molecular ladder (M) in kDa, FluPolB heterotrimer and hANP32A are indicated on the left of the gel. "IN" corresponds to the input.

(C) Superposition of size exclusion chromatography profiles of FluPolB bound to 5′ vRNA end (1-12) (solid line), and with hANP32A (dotted line), at 150 mM NaCl. The relative absorbance at 280 nm (mAU) is on the y-axis. The elution volume (ml) is on the x-axis, graduated every 50 µl. SDS-PAGE fractions 1 to 13 corresponds to the elution volume 1.0 ml - 1.65 ml.

(D) Mass photometry analysis of FluPolB bound to the 5′ vRNA end (1-12) at 150 mM NaCl. The determined masses in kDa of the main species are indicated.

(E) Mass photometry analysis of FluPolB bound to the 5′ vRNA end (1-12) with excess of hANP32A at 150 mM NaCl. The determined masses in kDa of the main species are indicated.

Source data are provided as a Source Data file (n=1-3 independent experiments).

SUPPLEMENTARY FIGURE 8

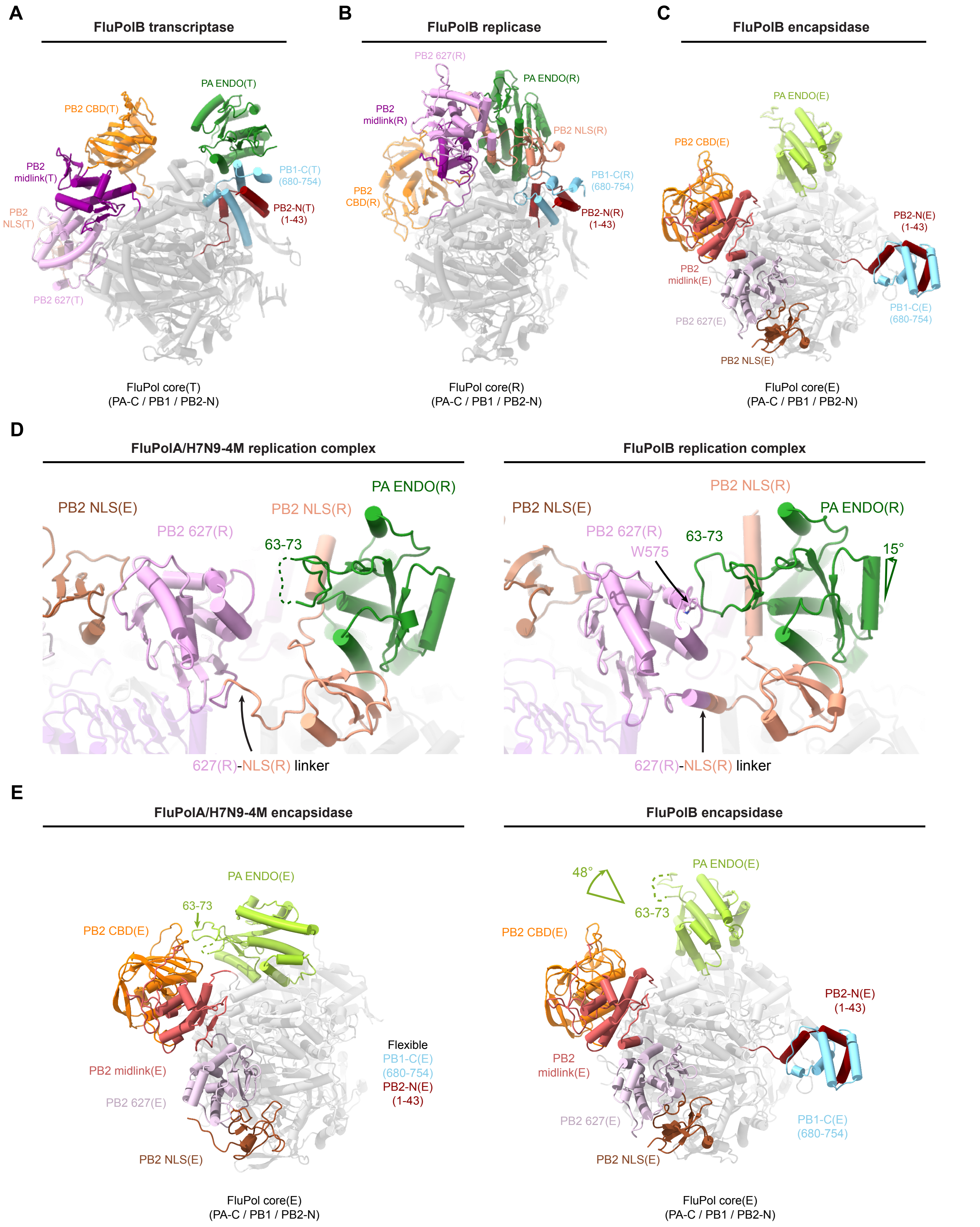

Supplementary Figure 8. Structural comparison of FluPolB transcriptase, replicase, encapsidase conformations and between FluA/H7N9-4M, FluB replication complexes.

(A) Cartoon representation of FluPolB in a transcriptase conformation (FluPolB(T)) (PDB 4WSA). FluPolB(T) core is dark grey, shown in transparency, PA ENDO(T) in dark green, PB1-C(T) in blue, PB2-N(T) in red, PB2 midlink(T) in magenta, PB2 CBD(T) in orange, PB2 627(T) in pink, PB2 NLS(T) in beige.

(B) Cartoon representation of FluPolB in a replicase conformation (FluPolB(R)), extracted from the replication complex and aligned on FluPolB(T) PB1 subunit. FluPolB(R) core is in dark grey, shown in transparency, PA ENDO(R) in dark green, PB1-C(R) in blue, PB2-N(R) in red, PB2 midlink(R) in magenta, PB2 CBD(R) in orange, PB2 627(R) in pink, PB2 NLS(R) in beige.

(C) Cartoon representation of FluPolB in an encapsidase conformation (FluPolB(E)), extracted from the replication complex and aligned on FluPolB(T) PB1 subunit. FluPolB(E) core is in light grey, shown in transparency, PA ENDO(E) in light green, PB1-C(R) in blue, PB2-N(R) in red, PB2 midlink(E) in salmon, PB2 CBD(E) in orange, PB2 627(E) in light pink, PB2 NLS(E) in brown. PB1-C(E) and PB2-N(E) helical bundle swung away, interacting with FluPolB(R) (shown in Supplementary Figure 9F).

(D) Structural comparison between FluA/H7N9-4M and FluB replication complexes. Domains are coloured as in (B-C). FluPolA/H7N9-4M PA ENDO(R) 63-73 loop is flexible, shown as a dotted line. FluPolB(R) equivalent interacts with FluPolB PB2 627(R), next to W575, displayed and indicated. FluPolB PA ENDO(R) compared to FluPolA/H7N9-4M PA ENDO(R) undergoes a 15 degree rotation, indicated with an arrow. PB2 627(R)/NLS(R) linkers are indicated, taking up an α-helical conformation in FluPolB replication complex.

(E) Structural comparison between FluPolA/H7N9-4M(E) and FluPolB(E). Domains are coloured as in (C). FluPolA/H7N9-4M PA ENDO(E) 63-73 insertion interacts with PB2 CBD(E) (as seen in Supplementary Figure 3F). FluPolB PA ENDO(E), compared to FluPolA/H7N9-4M PA ENDO(E), undergoes a 48 degree rotation, indicated an arrow. FluPolB PA ENDO(E) 63-73 loop is flexible, represented as a dotted line.

SUPPLEMENTARY FIGURE 9

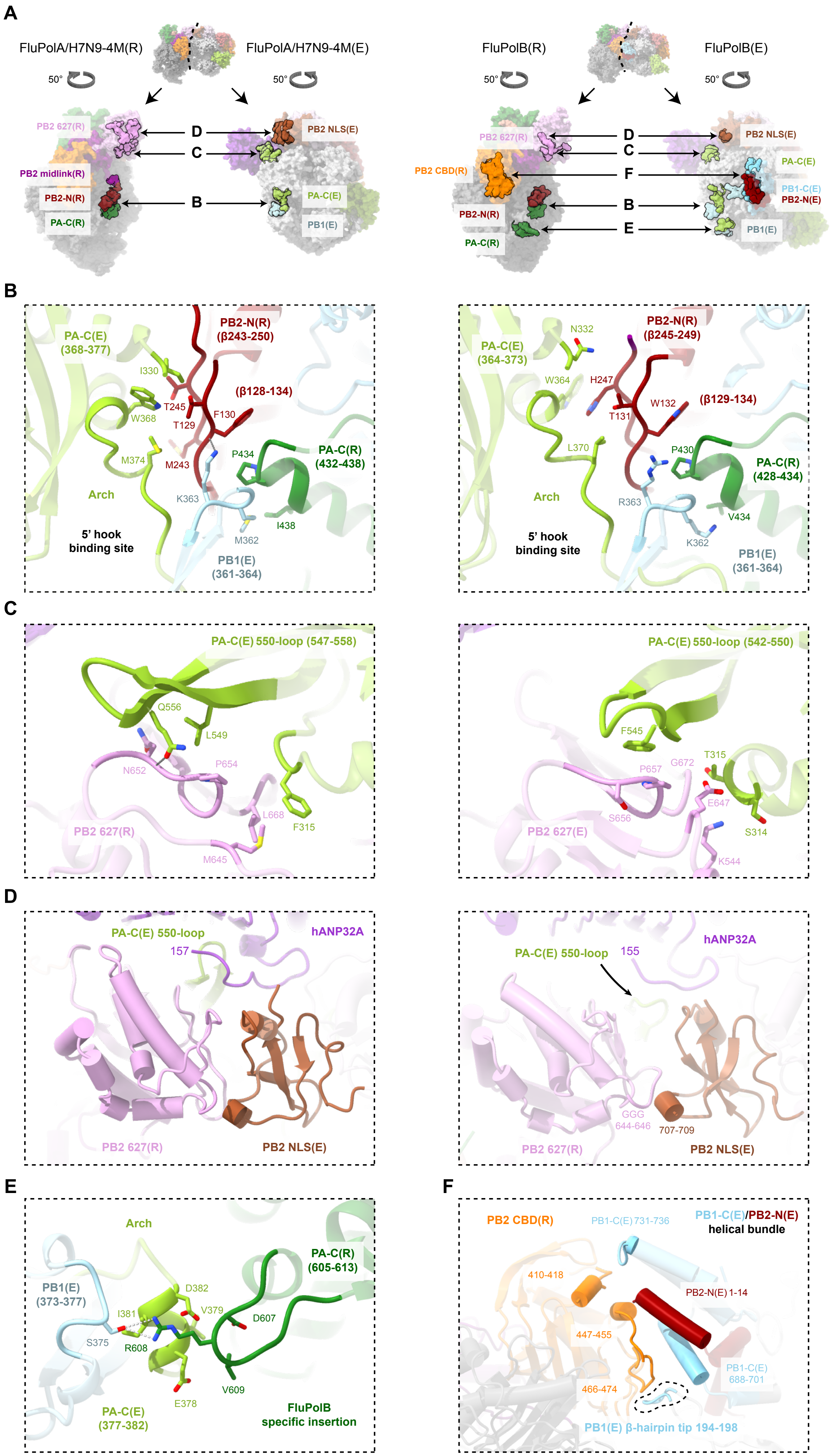

Supplementary Figure 9. Structural comparison between FluPolA/H7N9-4M and FluPolB replication complexes interfaces.

(A) Overview of the interacting domains between FluPols(R) and FluPols(E). Both replicase and encapsidase moiety were split and rotated by 50 degrees. Interacting surface is shown as non-transparent. Most domains are coloured as in Figure 2, with PA(R) in dark green, PB2-N(R) in red. For the three main conserved interfaces between FluPolA/H7N9-4M and FluPolB replication complexes, a close-up view is shown in panels (B), (C), (D).

(B) PA-C(R), PB2-N(R) and PA(E) arch interaction. Domains are coloured as in (A). Interacting residues are displayed, shown as non-transparent. Ionic and hydrogen bonds are shown as grey dotted lines.

(C) PB2 627(R) C-terminal β-sheet and PA-C 550-loop(E) interaction. Domains are coloured as in (A). Interacting residues are displayed, shown as non-transparent. Ionic and hydrogen bonds are shown as grey dotted lines.

(D) PB2 627(R) and PB2 NLS(E) interaction. Domains are coloured as in (A). Interacting residues are displayed, shown as non-transparent.

(E) FluPolB PA-C(R), PA-C(E) and PB1(E) specific interface. FluPolB PA-C(R) specific insertion 605-613 interacts with PA-C(E) 377-382 and PB1(E) 373-377. Domains are coloured as in (A). Interacting residues are displayed, shown as non-transparent.

(F) FluPolB PB1-C(E)/PB2-N(E) helical bundle interacts with PB2 CBD(R). Interacting Domains are coloured as in (A) and shown as non-transparent.

SUPPLEMENTARY FIGURE 10

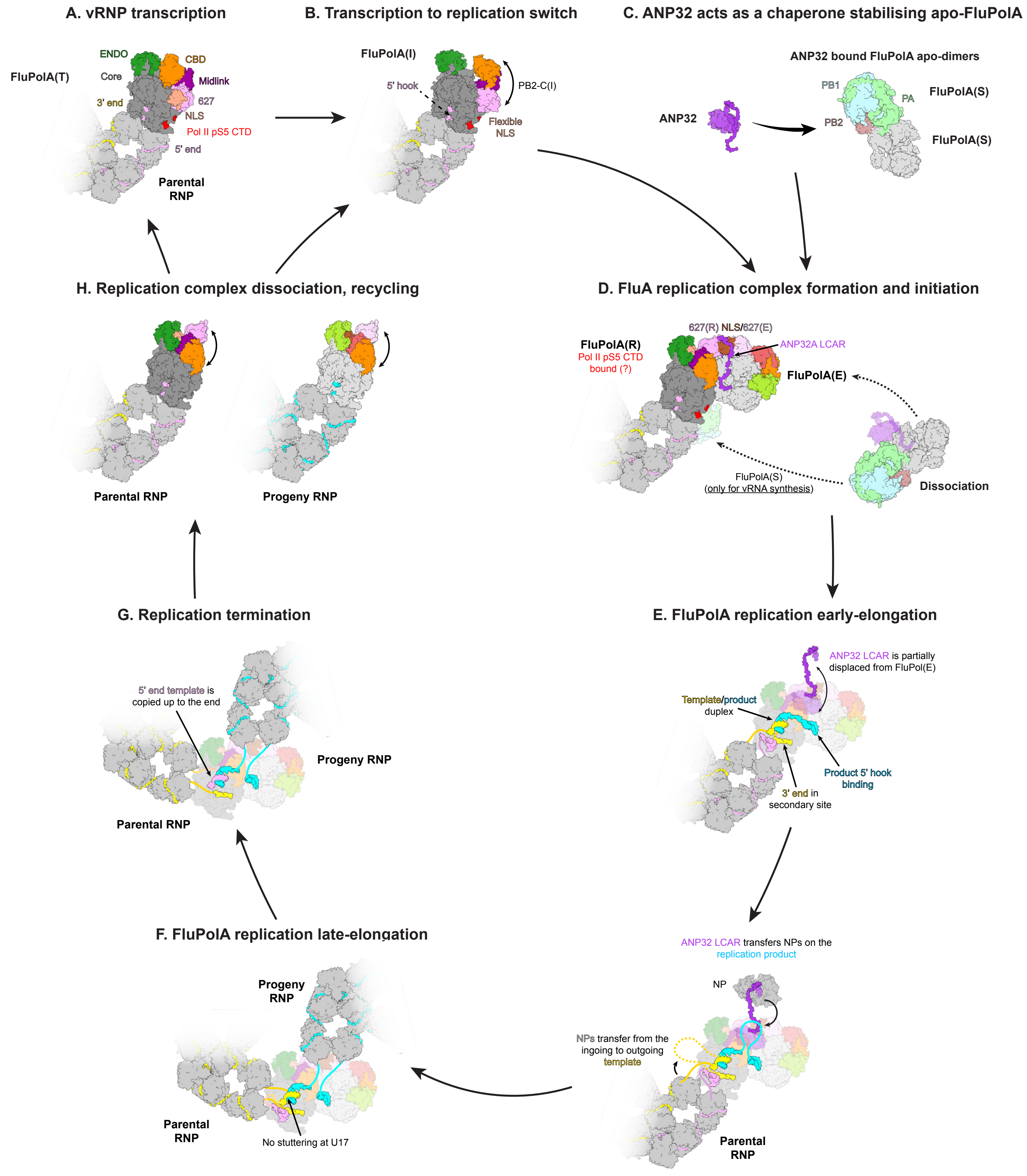

Supplementary Figure 10. Trimer model of FluA replication.

(A) vRNPs bound to cellular Pol II pS5 CTD (red) with the FluPol in the transcriptase conformation (FluPol(T)) perform transcription leading to the synthesis of capped/poly-adenylated viral mRNAs. These are translated by the host machinery to yield new viral proteins, including the apo-FluPols and NPs that are required for replication and are reimported into the nucleus.

(B) Within the parental vRNP, due to domain flexibility, the FluPol(T) conformation can transiently adopt the intermediate conformation FluPol(I)(Keown et al., 2022; Krischuns et al., 2024; Li et al., 2023).

(C) ANP32 acts as a chaperone at physiological salt concentration, stabilising apo-FluPol dimers through electrostatic interactions via the LCAR.

(D) Only in the presence of ANP32 bound apo-FluPol, possibly co-localised by interaction with the Pol II pS5 CTD, is the parental FluPol locked into the stable replicase conformation upon formation of the FluPol(R)-ANP32-FluPol(E) replication complex. The FluPol(E) must derive from dissociation of the symmetric dimer (which is in equilibrium with monomeric forms), bringing with it ANP32. While the ANP32 LCAR stabilises FluPol(E), the LRR domain bridges FluPol(E) to FluPol(R). Replication initiation may start concomitantly with replication complex formation. For cRNA synthesis, initiation is terminal but for vRNA synthesis, it is internal. For FluPolA, one FluPol from the apo-dimer (FluPolA(S)) is proposed to form a symmetric dimer with FluPol(R), allowing template realignment in the case of vRNA synthesis (Fan et al., 2019).

(E) FluPol(R) synthesises the complementary replication product. In early elongation, the newly synthetized 5' end binds to the FluPol(E) hook binding site, accessible from the replicase through a protected channel. Simultaneously, the ANP32 LCAR, where may initially prevent non-specific RNA binding, is partially displaced from FluPol(E). It becomes available for recruiting apo-NPs that successively will bind the replication product bulging out of the replication complex. Concomitantly, NPs are transferred from the ingoing to outgoing template in the parental replicase RNP.

(F) FluPol(R) processively copies the template until it reaches nucleotide 17 from the 5' end whereupon further template translocation is normally resisted by the tight binding of the 5' hook. In the case of transcription of the vRNA template, this leads to poly-adenylation through repeated copying of U17. However, in replication, FluPol(R) manages to read-through to the end of the template. At this stage, a progeny nucleocapsid has formed, packaging the almost full-length replication product.

(G) Replication termination. To synthetize a full-length complementary replication product, the 5' end of the template must be released from its binding site and fully copied to its extremity. The mechanism for this is unknown.

(H) Replication complex dissociation and recycling. Once replication is terminated, FluPol recycling can occur as previously proposed for the transcription cycle (Wandzik et al., 2020). FluPol(R), within the parental RNP, can perform another round of replication (or potentially transcription). The erstwhile FluPol(E), now part of the progeny RNP, can become a replicase or a secondary transcriptase.

SUPPLEMENTARY FIGURE 11

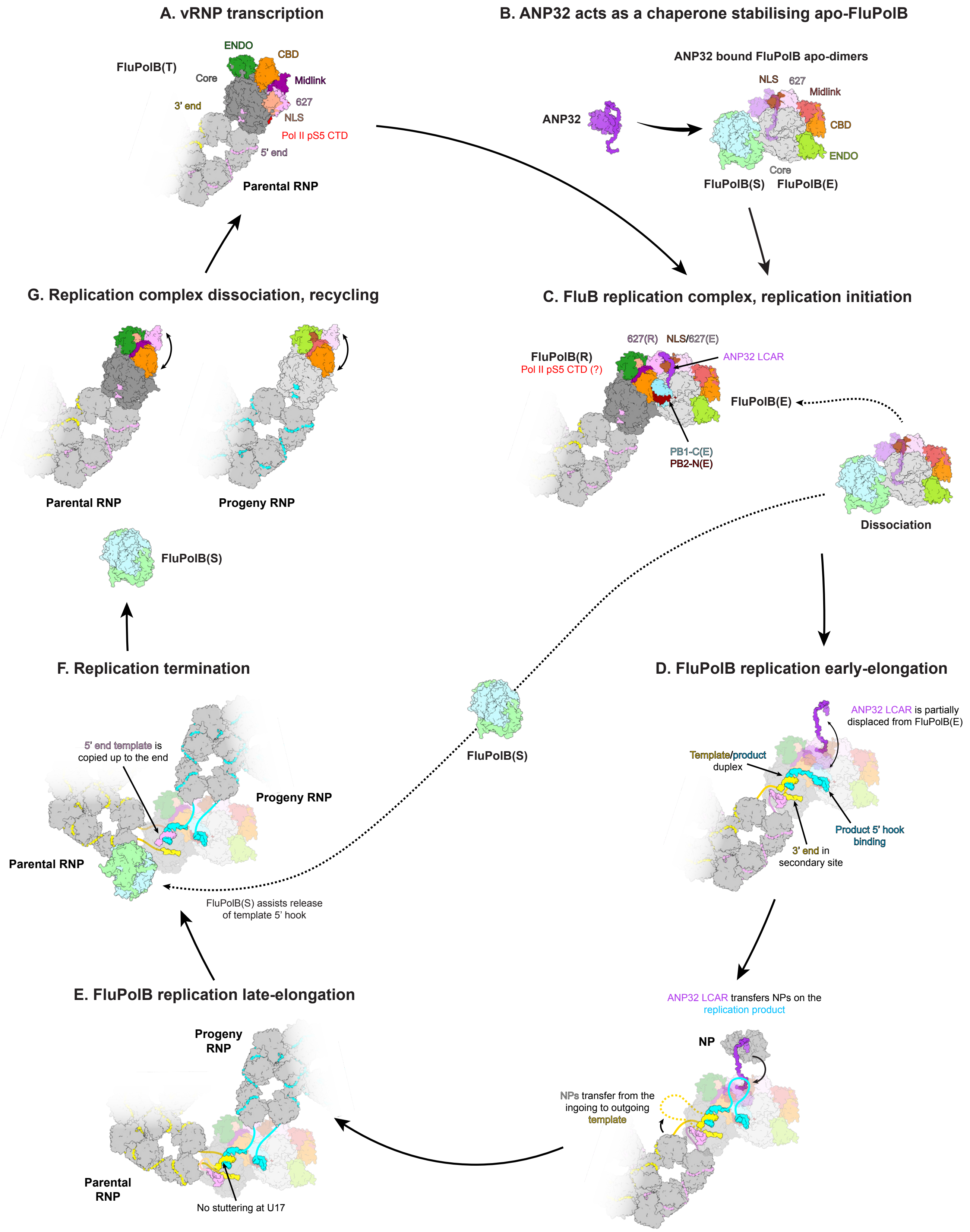

Supplementary Figure 11. Trimer model of FluB replication.

Similar to Supplementary Figure 10, but omitting steps not yet shown for FluB.
In (F), based on the FluB trimeric replication complex structure, it is suggested that a FluPol(S) from the apo-FluPolB dimer could interact with FluPolB(R), at a late stage in replication, forcing release of the 5' hook and stabilising the replicase while it copies the last nucleotides up to the template 5' end.

SUPPLEMENTARY NOTE 1

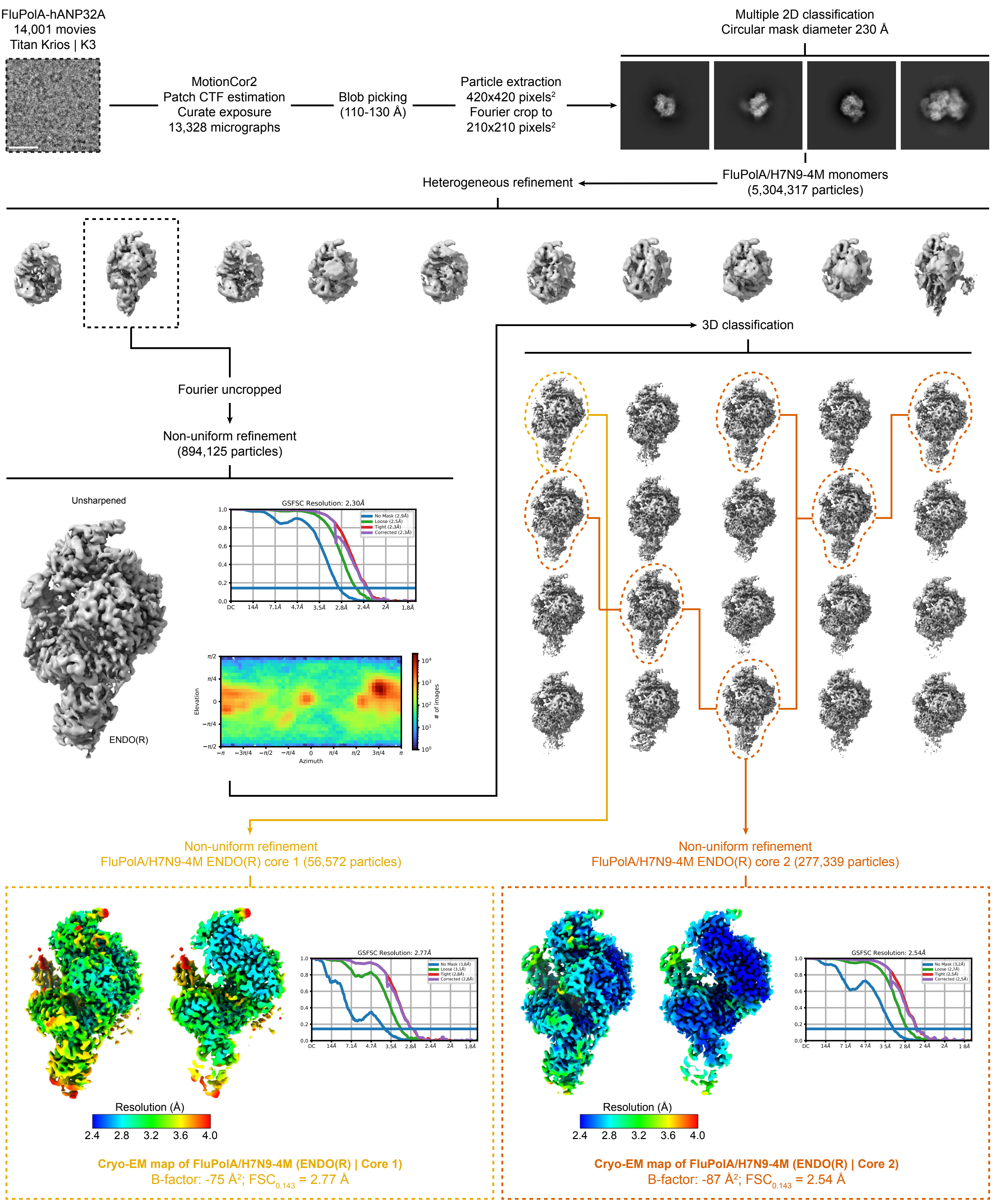

Supplementary Note 1. Cryo-EM image processing strategy applied to obtain FluPolA/H7N9-4M ENDO(R) core1 and core2 structures.

Schematics of the image processing strategy used with the data collected on a TEM Titan Krios equipped with a Gatan K3 direct electron detector mounted on a Gatan Bioquantum energy filter. Representative cropped micrograph, 2D class averages and 3D classes are displayed. Full and cutaway views of each local resolution filtered EM maps are shown. Fourier shell correlation curves (FSC) are displayed. Scale bar = 200 Å.

SUPPLEMENTARY NOTE 2

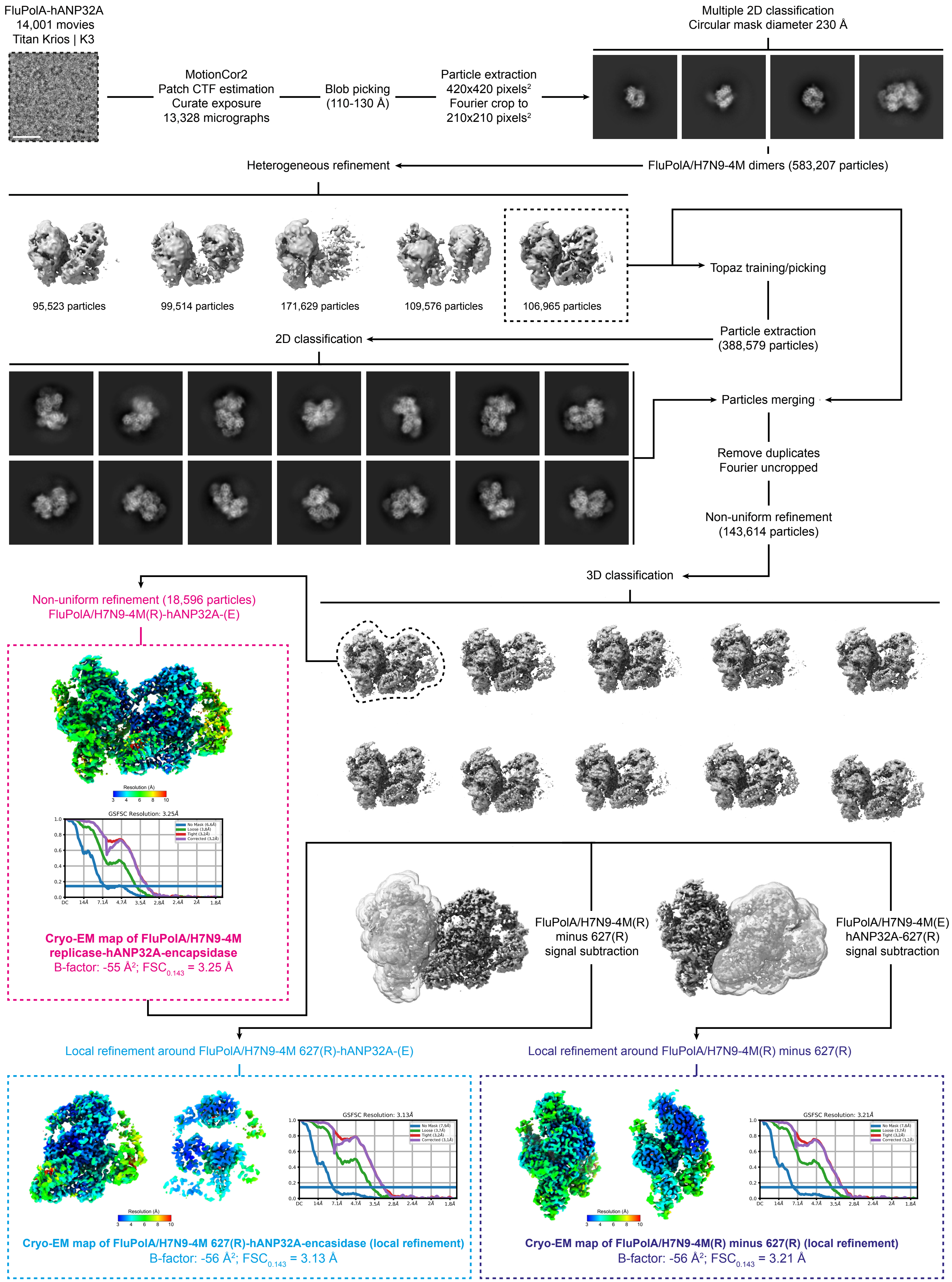

Supplementary Note 2. Cryo-EM image processing strategy applied to obtain FluA replication complex structures.

Schematics of the image processing strategy used with the data collected on a TEM Titan Krios equipped with a Gatan K3 direct electron detector mounted on a Gatan Bioquantum energy filter. Representative cropped micrograph, 2D class averages and 3D classes are displayed. Full and cutaway views of each DeepEMhancer (Sanchez-Garcia et al., 2021) filtered EM maps are shown. Fourier shell correlation curves (FSC) are displayed. Scale bar = 200 Å.

SUPPLEMENTARY NOTE 3

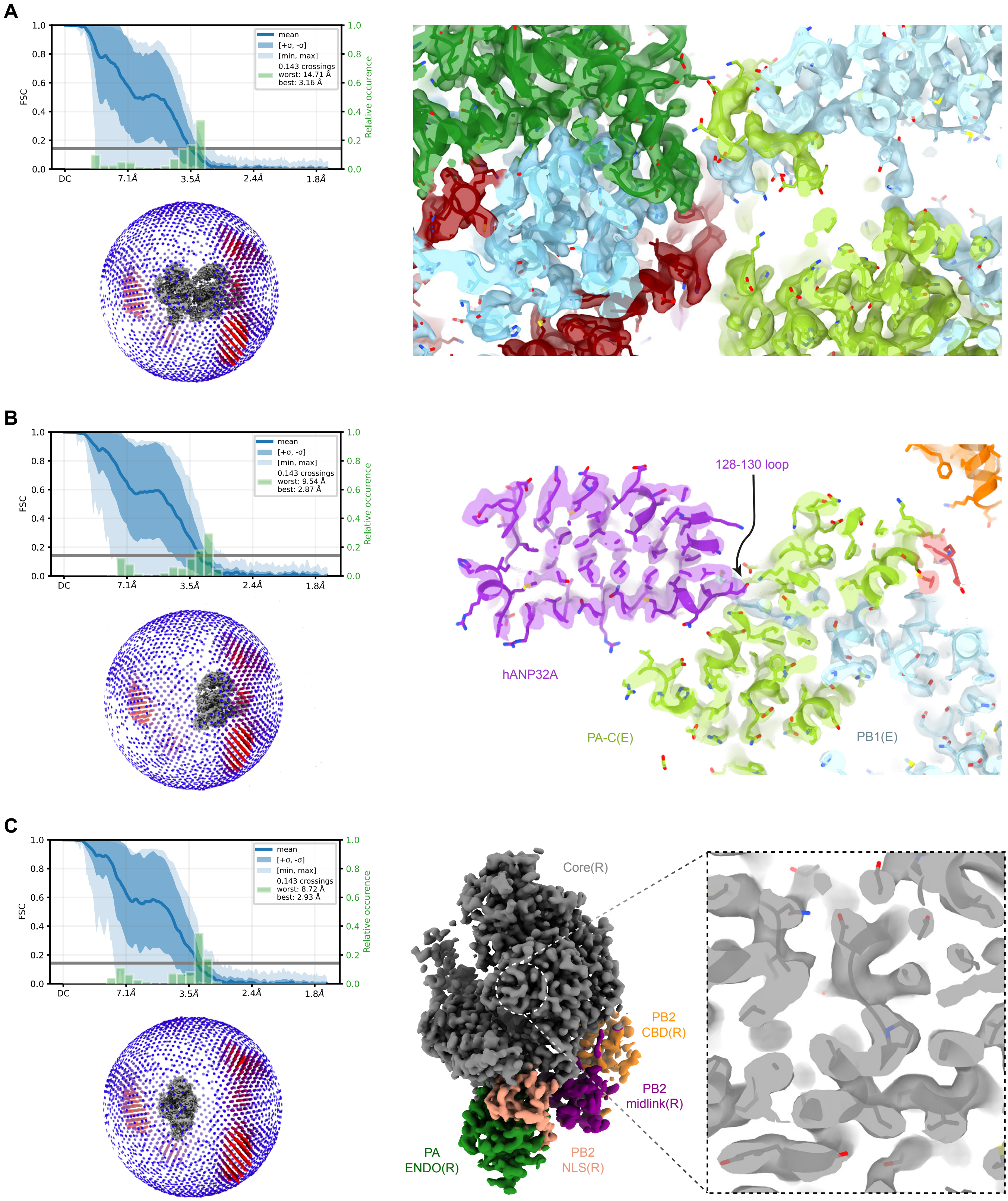

Supplementary Note 3. 3D-FSCs, orientation distribution and cryo-EM map quality of FluA replication complex structures.

(A) 3D-FSC from the complete FluA replication complex map, particle distribution plot, and corresponding map cut-away view of FluPolA replicase-encapsidase interface. Domains are coloured as in Supplementary Figure 4. Side chains are displayed and coloured by hetero-atoms.

(B) 3D-FSC from the local refinement map around hANP32A and FluPolA encapsidase, particle distribution plot, and corresponding map cut-away view focused on hANP32A 128-130 loop with FluPolA encapsidase. Domains are coloured as in Fig.2. Side chains are displayed and coloured by hetero-atoms.

(C) 3D-FSC from the local refinement map around FluPolA replicase, particle distribution plot, and corresponding map with a closed-up view. Domains are coloured as in Fig.2. Side chains are displayed and coloured by hetero-atoms.

SUPPLEMENTARY NOTE 4

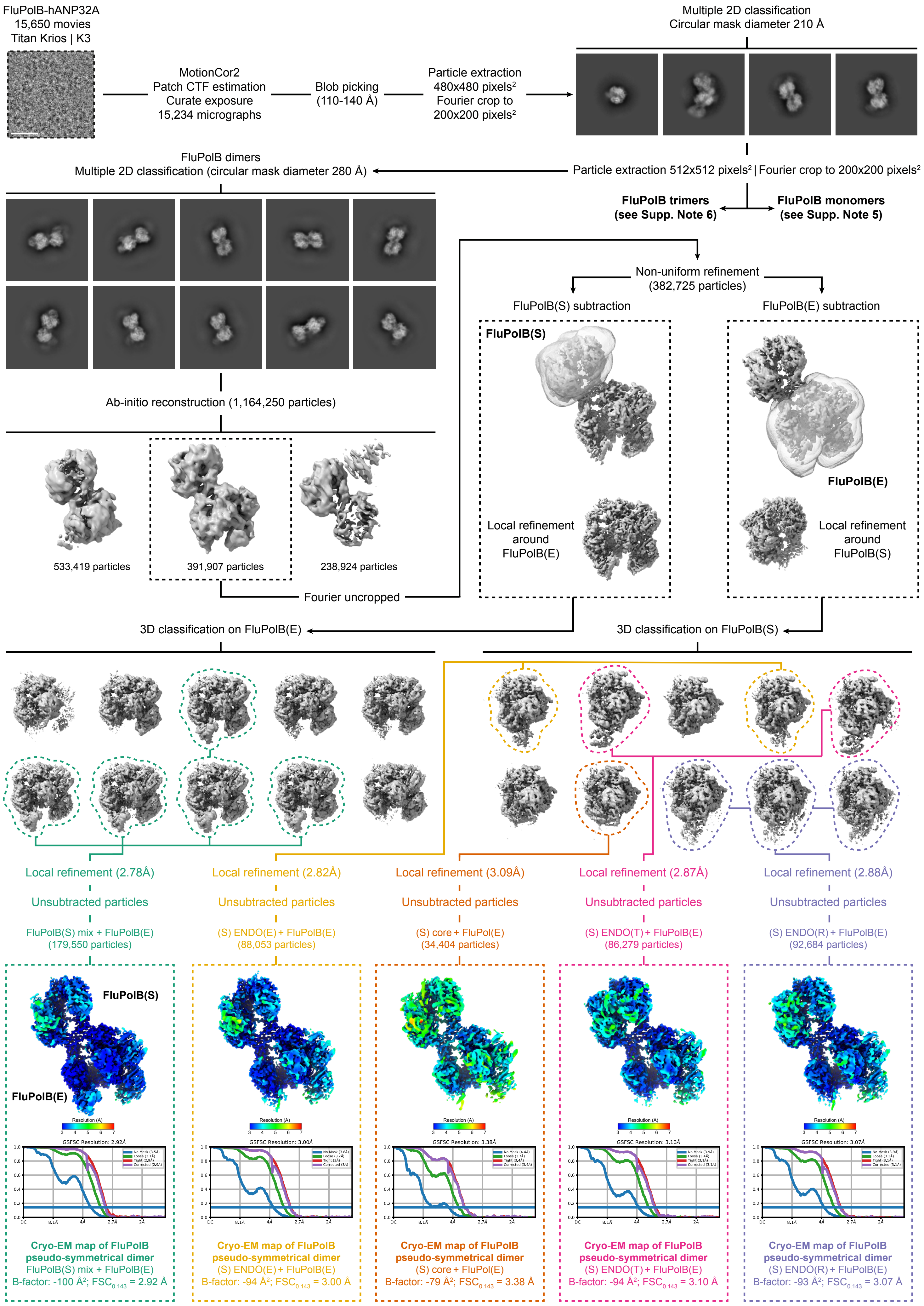

Supplementary Note 4. Cryo-EM image processing strategy applied to obtain FluPolB pseudo-symmetrical dimer structures, with one moiety being an encapsidase.

Schematics of the image processing strategy used with the data collected on a TEM Titan Krios equipped with a Gatan K3 direct electron detector mounted on a Gatan Bioquantum energy filter. Representative cropped micrograph, 2D class averages and 3D classes are displayed. Full and cutaway views of each local resolution filtered EM maps are shown. Fourier shell correlation curves (FSC) are displayed. Scale bar = 200 Å.

SUPPLEMENTARY NOTE 5

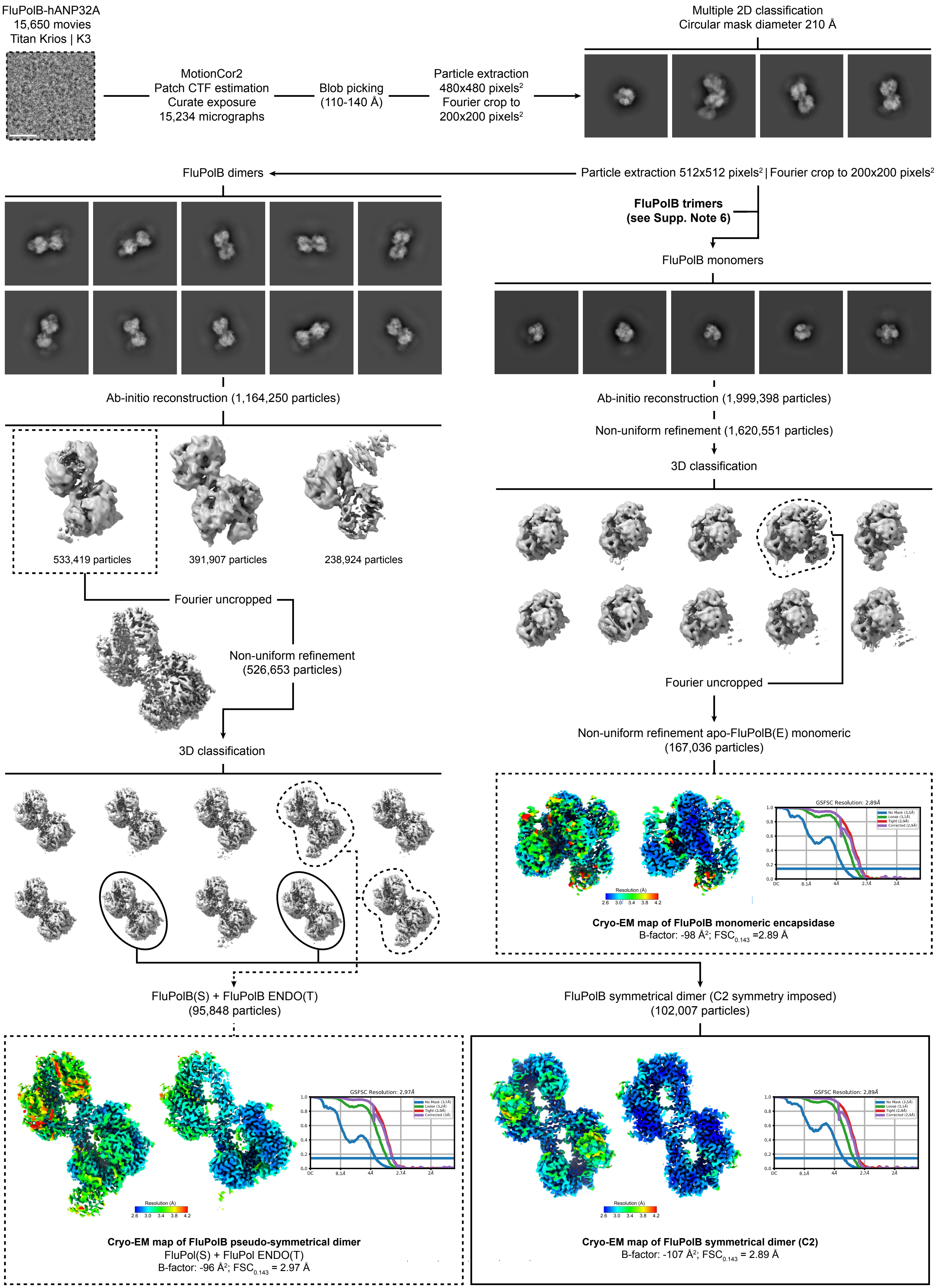

Supplementary Note 5. Cryo-EM image processing strategy applied to obtain FluPolB (pseudo-)symmetrical dimer structures and monomeric apo-FluPolB encapsidase.

Schematics of the image processing strategy used with the data collected on a TEM Titan Krios equipped with a Gatan K3 direct electron detector mounted on a Gatan Bioquantum energy filter. Representative cropped micrograph, 2D class averages and 3D classes are displayed. Full and cutaway views of each local resolution filtered EM maps are shown. Fourier shell correlation curves (FSC) are displayed. Scale bar = 200 Å.

SUPPLEMENTARY NOTE 6

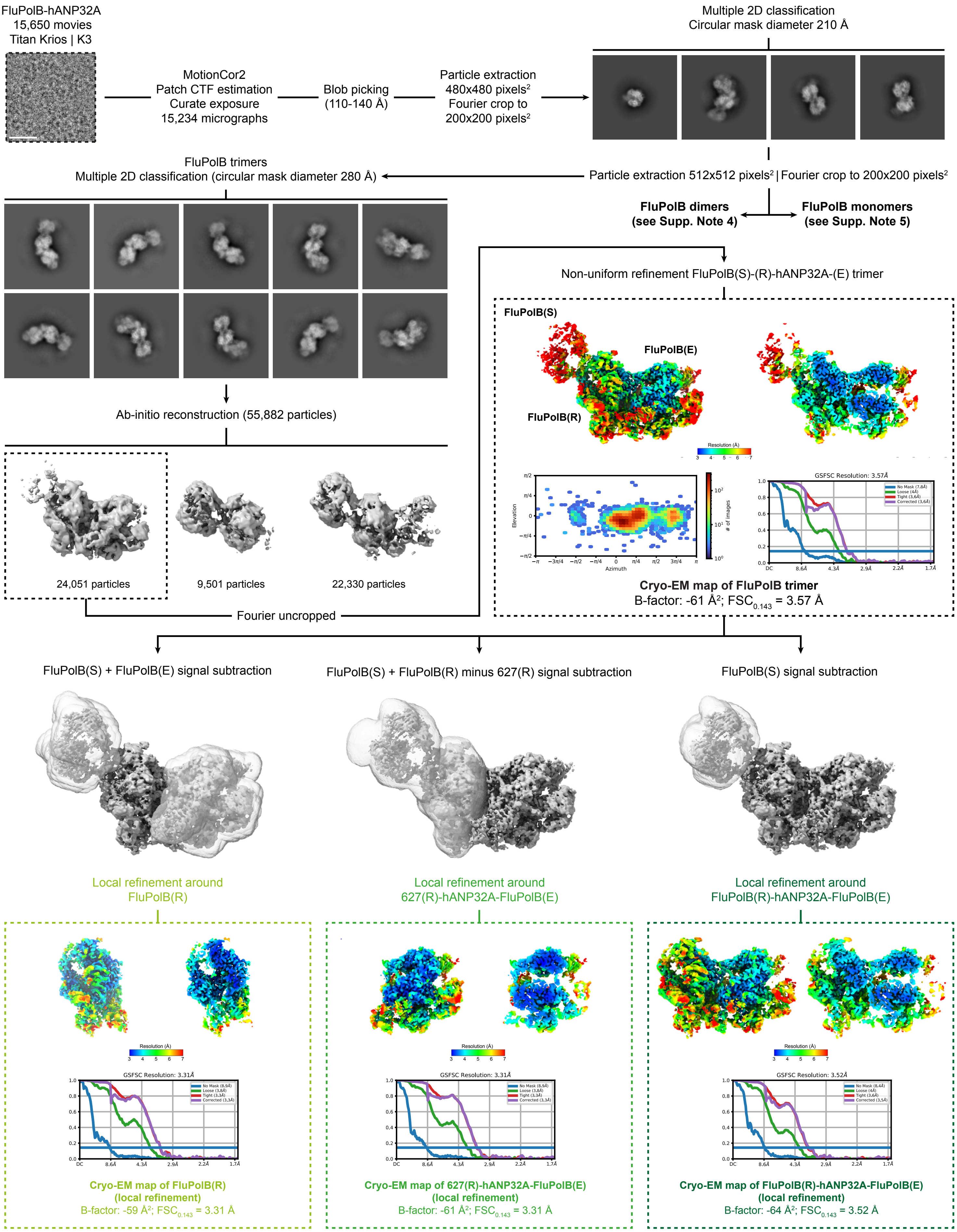

Supplementary Note 6. Cryo-EM image processing strategy applied to obtain FluPolB replication complex structures.

Schematics of the image processing strategy used with the data collected on a TEM Titan Krios equipped with a Gatan K3 direct electron detector mounted on a Gatan Bioquantum energy filter. Representative cropped micrograph, 2D class averages and 3D classes are displayed. Full and cutaway views of each DeepEMhancer (Sanchez-Garcia et al., 2021) filtered EM maps are shown. Fourier shell correlation curves (FSC) are displayed. Scale bar = 200 Å.

SUPPLEMENTARY NOTE 7

A

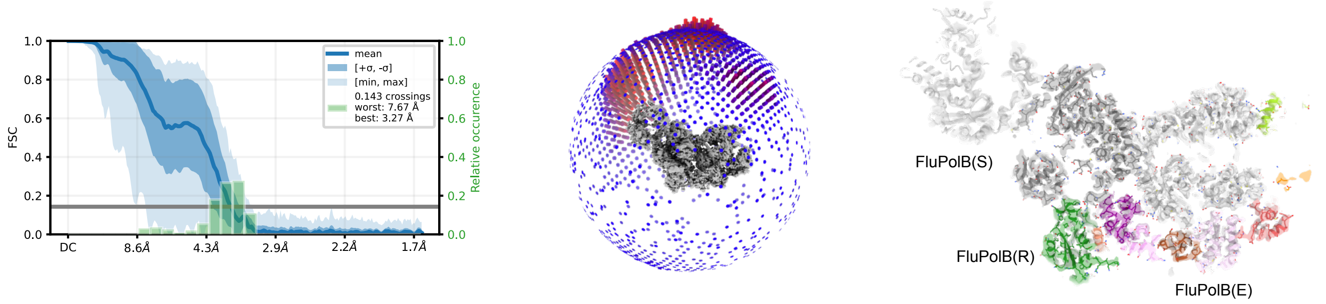

B

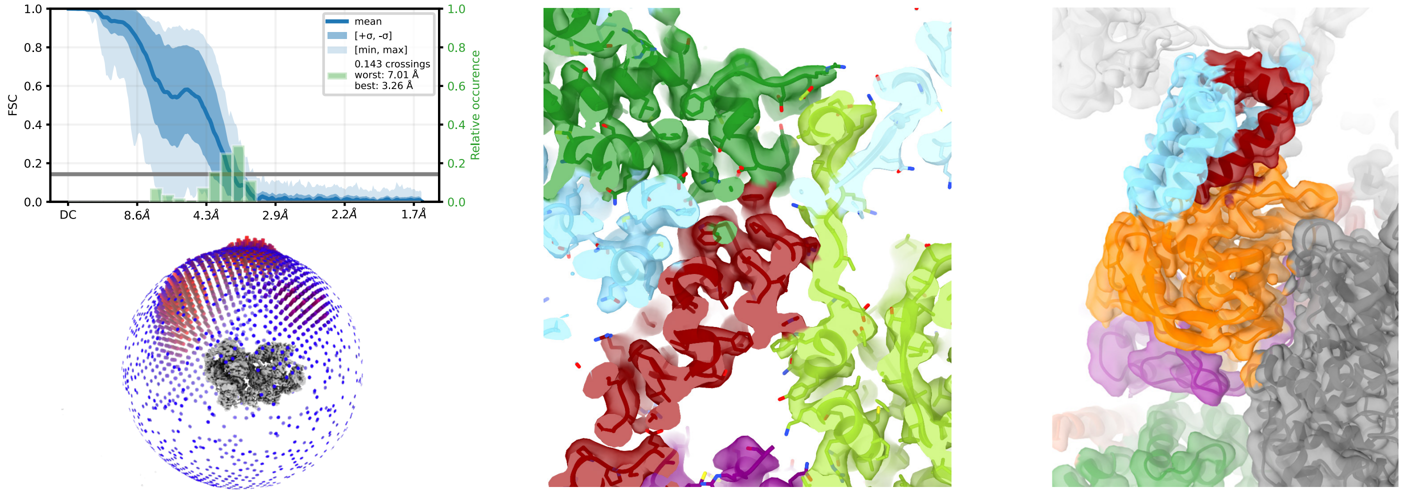

C

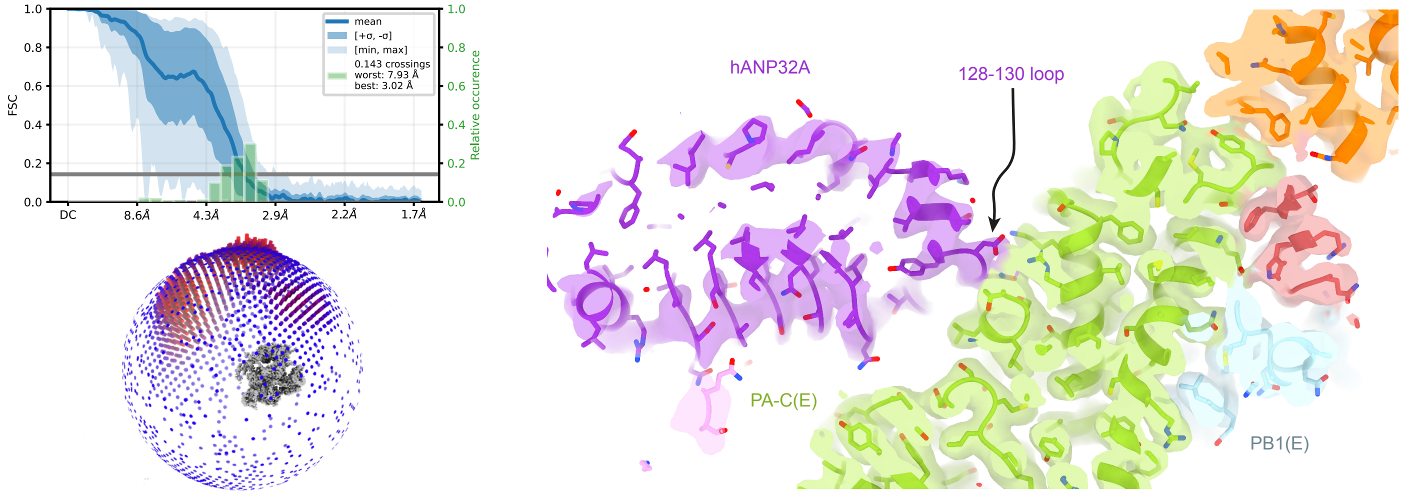

D

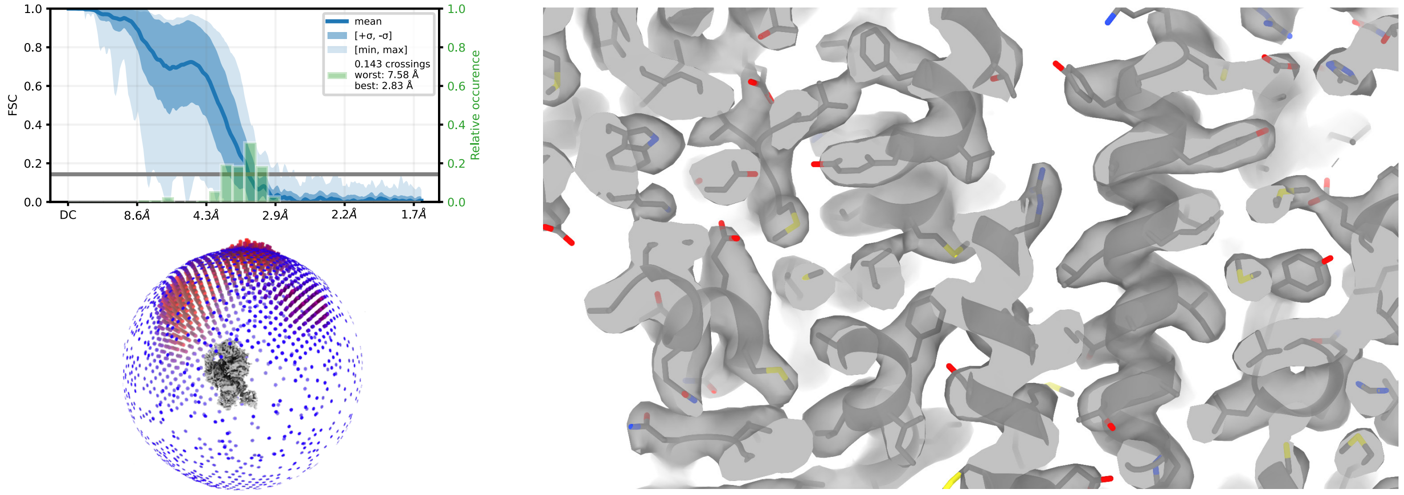

Supplementary Note 7. 3D-FSCs, orientation distribution and cryo-EM map quality of FluB replication complex structures.

(A) 3D-FSC from the complete FluB trimer map, particle distribution plot, and map cut-away view coloured as in Fig.5 with side chains displayed and coloured by hetero-atoms.

(B) 3D-FSC from the local refinement map around FluB replication complex, particle distribution plot, and corresponding map cut-away view of FluPolB replicase-encapsidase interfaces coloured as in Supplementary Figure 9B, F. Side chains are displayed and coloured by hetero-atoms (right panel).

(C) 3D-FSC from the local refinement map around hANP32A and FluPolB encapsidase, particle distribution plot, and corresponding map cut-away view focused on hANP32A 128-130 loop with FluPolB encapsidase. Domains are coloured as in Fig.5. Side chains are displayed and coloured by hetero-atoms.

(D) 3D-FSC from the local refinement map around FluPolB replicase, particle distribution plot, and corresponding map with a closed-up view. Domains are coloured as in Fig.5. Side chains are displayed and coloured by hetero-atoms.

SUPPLEMENTARY NOTE 8

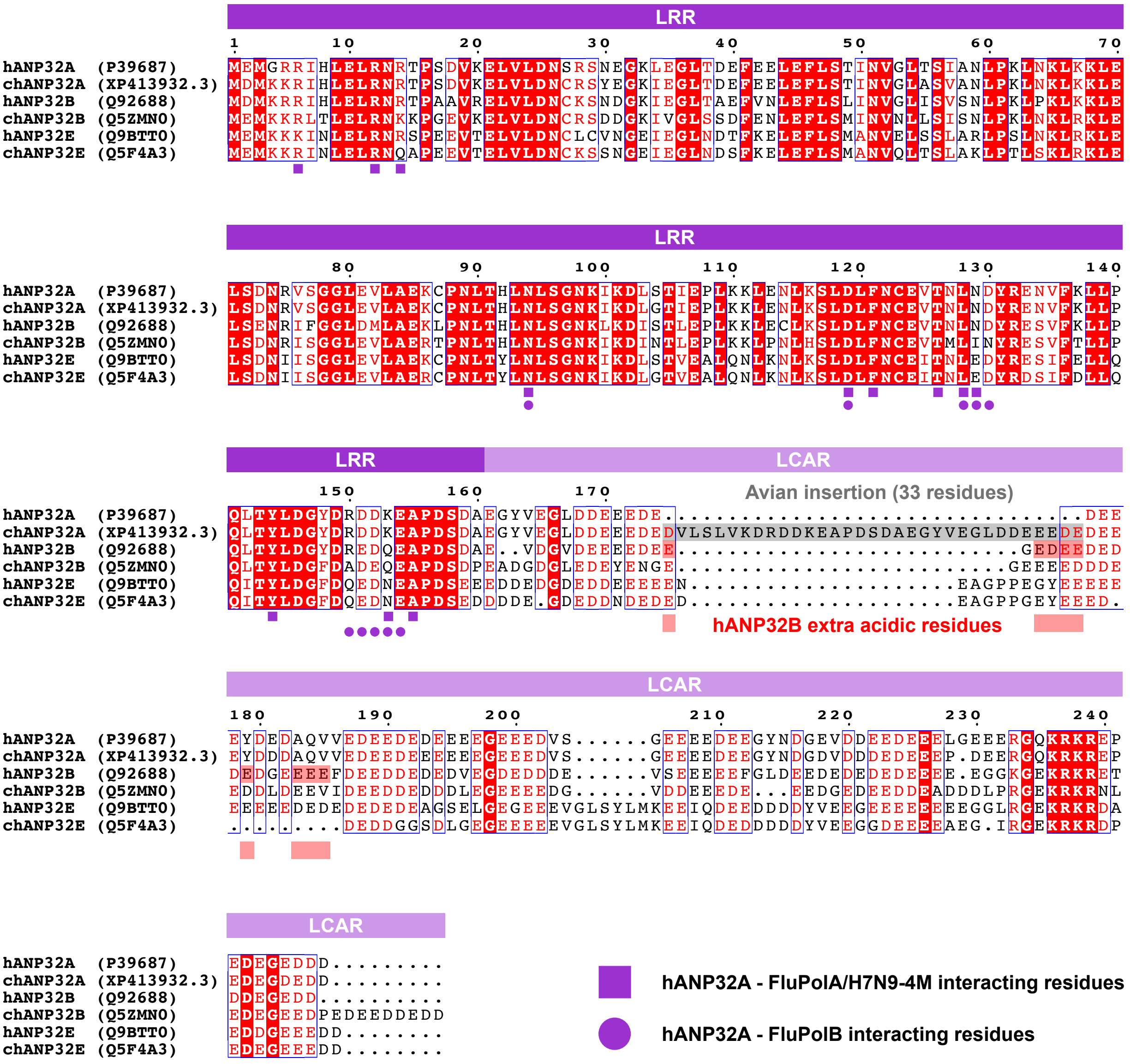

Supplementary Note 8. Multiple sequence alignment of human and chicken ANP32A, B and E.

Human (h) and chicken (ch) ANP32A/B/E UniProt numbers are indicated. Both Leucine Rich Repeat (LRR) and Low Complexity Acidic Region (LCAR) are indicated on top of the aligned sequences, respectively in dark and light purple. hANP32A interacting residues with FluPolA/H7N9-4M and FluPolB are indicated below aligned sequences, with respectively purple squares, or circles. The specific avian insertion of 33 residues is highlighted with a grey rectangle. hANP32B extra acidic residues are highlighted with red rectangles.

SUPPLEMENTARY NOTE 9

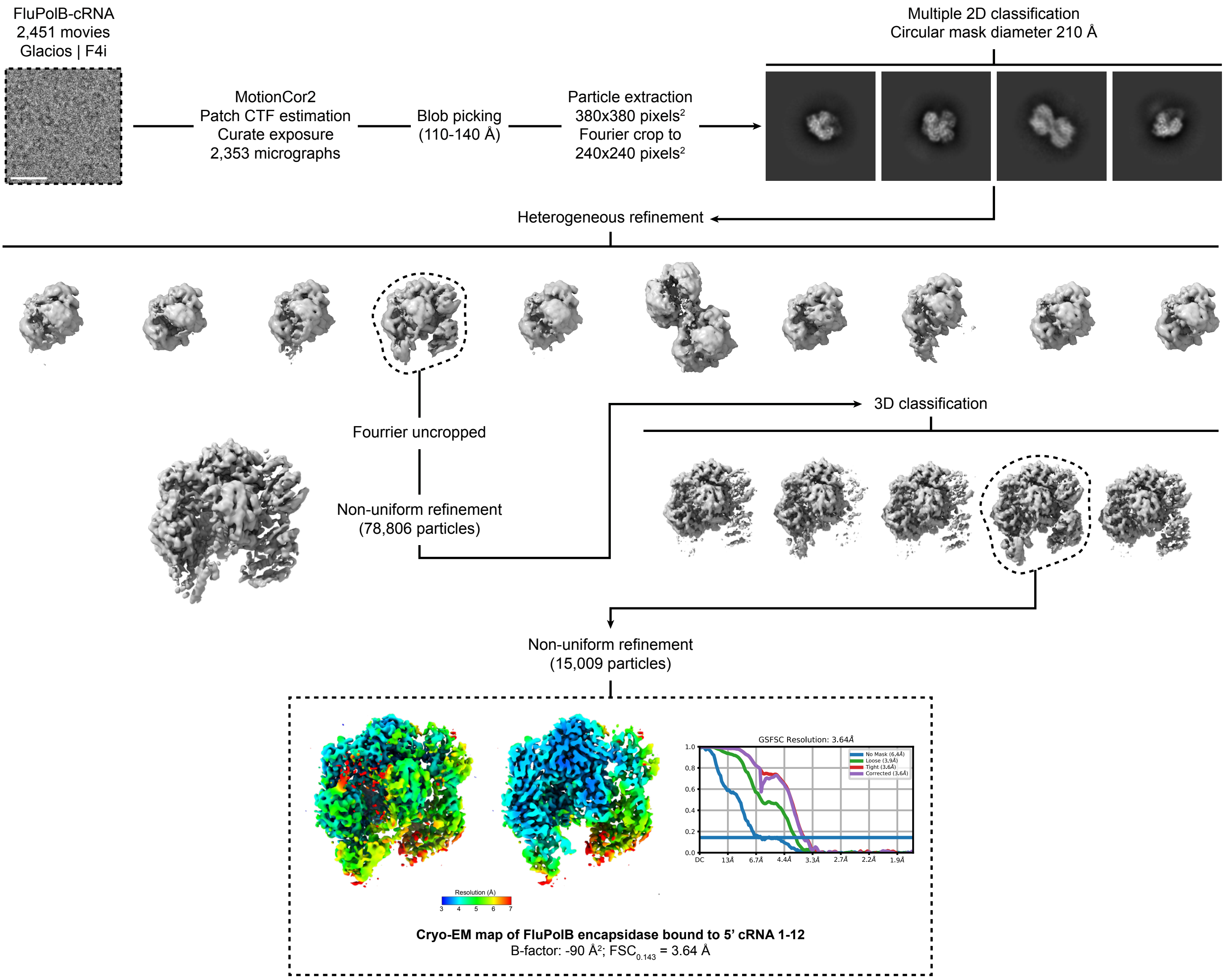

Supplementary Note 9. Cryo-EM image processing strategy applied to obtain FluPolB encapsidase bound to 5' cRNA 1-12.

Schematics of the image processing strategy used with the data collected on a TEM Glacios equipped with a Falcon4i direct electron detector mounted on a SelectrisX energy filter. Representative cropped micrograph, 2D class averages and 3D classes are displayed. Full and cutaway views of each local resolution filtered EM maps are shown. Fourier shell correlation curves (FSC) are displayed. Scale bar = 200 Å.

Supplementary References

1. Fan, H., Walker, A.P., Carrique, L., Keown, J.R., Serna Martin, I., Karia, D., Sharps, J., Hengrung, N., Pardon, E., Steyaert, J., et al. (2019). Structures of influenza A virus RNA polymerase offer insight into viral genome replication. Nature 573, 287-290.

2. Keown, J.R., Zhu, Z., Carrique, L., Fan, H., Walker, A.P., Serna Martin, I., Pardon, E., Steyaert, J., Fodor, E., and Grimes, J.M. (2022). Mapping inhibitory sites on the RNA polymerase of the 1918 pandemic influenza virus using nanobodies. Nat Commun 13, 251.

3. Krischuns, T., Arragain, B., Isel, C., Paisant, S., Budt, M., Wolff, T., Cusack, S., and Naffakh, N. (2024). The host RNA polymerase II C-terminal domain is the anchor for replication of the influenza virus genome. Nat Commun 15, 1064.

4. Li, H., Wu, Y., Li, M., Guo, L., Gao, Y., Wang, Q., Zhang, J., Lai, Z., Zhang, X., Zhu, L., et al. (2023). An intermediate state allows influenza polymerase to switch smoothly between transcription and replication cycles. Nature structural & molecular biology 30, 1183-1192.

5. Sanchez-Garcia, R., Gomez-Blanco, J., Cuervo, A., Carazo, J.M., Sorzano, C.O.S., and Vargas, J. (2021). DeepEMhancer: a deep learning solution for cryo-EM volume post-processing. Commun Biol 4, 874.

6. Wandzik, J.M., Kouba, T., Drncova, P., Karuppasamy, M., Pflug, A., Provaznik, J., Azevedo, N., and Cusack, S. (2020). A structure-based model for the complete transcription cycle of influenza polymerase. Cell.
