# Supplementary material for: Structures of influenza A and B replication complexes give insight into avian to human host adaptation and reveal a role of ANP32 as an electrostatic chaperone for the apo-polymerase
Source: Nat Commun. 2024 Aug 19;15:6910. doi: 10.1038/s41467-024-51007-3 (PMC11333492; doi:10.1038/s41467-024-51007-3)
Supplement: Supplementary file 3 — Reporting Summary [file 41467_2024_51007_MOESM3_ESM.pdf]

nature portfolio

Corresponding author(s): Stephen Cusack

Last updated by author(s): 15/02/2024

Reporting Summary

Nature Portfolio wishes to improve the reproducibility of the work that we publish. This form provides structure for consistency and transparency in reporting. For further information on Nature Portfolio policies, see our Editorial Policies and the Editorial Policy Checklist.

Statistics

For all statistical analyses, confirm that the following items are present in the figure legend, table legend, main text, or Methods section.

n/a	Confirmed	
☐	☒	The exact sample size (*n*) for each experimental group/condition, given as a discrete number and unit of measurement
☐	☒	A statement on whether measurements were taken from distinct samples or whether the same sample was measured repeatedly
☐	☒	The statistical test(s) used AND whether they are one- or two-sided<br>*Only common tests should be described solely by name; describe more complex techniques in the Methods section.*
☒	☐	A description of all covariates tested
☐	☒	A description of any assumptions or corrections, such as tests of normality and adjustment for multiple comparisons
☐	☒	A full description of the statistical parameters including central tendency (e.g. means) or other basic estimates (e.g. regression coefficient) AND variation (e.g. standard deviation) or associated estimates of uncertainty (e.g. confidence intervals)
☐	☒	For null hypothesis testing, the test statistic (e.g. *F*, *t*, *r*) with confidence intervals, effect sizes, degrees of freedom and *P* value noted<br>*Give P values as exact values whenever suitable.*
☒	☐	For Bayesian analysis, information on the choice of priors and Markov chain Monte Carlo settings
☒	☐	For hierarchical and complex designs, identification of the appropriate level for tests and full reporting of outcomes
☒	☐	Estimates of effect sizes (e.g. Cohen's *d*, Pearson's *r*), indicating how they were calculated

Our web collection on statistics for biologists contains articles on many of the points above.

Software and code

Policy information about availability of computer code

Data collection	EPU
Data analysis	cryoSPARC v4.3.1 ; Relion 4.0.1 ; Phenix version 1.21.1-5286-000

For manuscripts utilizing custom algorithms or software that are central to the research but not yet described in published literature, software must be made available to editors and reviewers. We strongly encourage code deposition in a community repository (e.g. GitHub). See the Nature Portfolio guidelines for submitting code & software for further information.

Data

Policy information about availability of data

All manuscripts must include a data availability statement. This statement should provide the following information, where applicable:

- Accession codes, unique identifiers, or web links for publicly available datasets
- A description of any restrictions on data availability
- For clinical datasets or third party data, please ensure that the statement adheres to our policy

Influenza polymerase A/H7N9-4M (ENDO(R) | Core1) PDB ID 8RMP, EMD-19366;
Influenza polymerase A/H7N9-4M (ENDO(R) | Core2) PDB ID 8RMQ, EMD-19367;
Influenza polymerase A/H7N9-4M replication complex, an asymmetric polymerase dimer bound to human ANP32A PDB ID 8RMR, EMD-19368;
Influenza polymerase A/H7N9-4M replicase minus 627(R) (from "Influenza polymerase A/H7N9-4M replication complex" | Local refinement) PDB ID 8RMS,

EMD-19369;
Influenza polymerase A/H7N9-4M encapsidase plus 627(R) / human ANP32A (from "Influenza polymerase A/H7N9-4M replication complex" | Local refinement) PDB ID 8RN0, EMD-19382;
Influenza B polymerase, monomeric encapsidase with 5' cRNA hook bound PDB ID 8RN1, EMD-19383;
Monomeric apo-influenza B polymerase, encapsidase conformation PDB ID 8RN2, EMD-19384;
Pseudo-symmetrical influenza B polymerase apo-dimer, encapsidase moiety (from "Influenza B polymerase pseudo-symmetrical dimer" | Local refinement) PDB ID 8RN3, EMD-19385;
Pseudo-symmetrical influenza B polymerase apo-dimer, ENDO(T) moiety (from "Influenza B polymerase pseudo-symmetrical dimer" | Local refinement) PDB ID 8RN4, EMD-19386;
Pseudo-symmetrical influenza B polymerase apo-dimer, ENDO(R) moiety (from "Influenza B polymerase pseudo-symmetrical dimer" | Local refinement) PDB ID 8RN5, EMD-19387;
Pseudo-symmetrical influenza B polymerase apo-dimer, ENDO(E) moiety (from "Influenza B polymerase pseudo-symmetrical dimer" | Local refinement) PDB ID 8RN6, EMD-19388;
Pseudo-symmetrical influenza B polymerase apo-dimer, core-only moiety (from "Influenza B polymerase pseudo-symmetrical dimer" | Local refinement) PDB ID 8RN7, EMD-19389;
Influenza B polymerase pseudo-symmetrical apo-dimer (FluPol(E)|FluPol(S)) PDB ID 8RN8, EMD-19390;
Influenza B polymerase, replicase (from "Influenza B polymerase apo-trimer" | Local refinement) PDB ID 8RN9, EMD-19391;
Influenza B polymerase, encapsidase plus 627(R) / human ANP32A (from "Influenza B polymerase apo-trimer" | Local refinement) PDB ID 8RNB, EMD-19393;
Influenza B polymerase, replication complex, an asymmetric polymerase dimer bound to human ANP32A (from "Influenza B polymerase apo-trimer" | Local refinement) PDB ID 8RNC, EMD-19394;
Influenza B polymerase apo-trimer PDB ID 8RNA, EMD-19392.

Research involving human participants, their data, or biological material

Policy information about studies with human participants or human data. See also policy information about sex, gender (identity/presentation), and sexual orientation and race, ethnicity and racism.

Reporting on sex and gender	N/A
Reporting on race, ethnicity, or other socially relevant groupings	N/A
Population characteristics	N/A
Recruitment	N/A
Ethics oversight	N/A

Note that full information on the approval of the study protocol must also be provided in the manuscript.

Field-specific reporting

Please select the one below that is the best fit for your research. If you are not sure, read the appropriate sections before making your selection.

☒ Life sciences ☐ Behavioural & social sciences ☐ Ecological, evolutionary & environmental sciences

For a reference copy of the document with all sections, see nature.com/documents/nr-reporting-summary-flat.pdf

Life sciences study design

All studies must disclose on these points even when the disclosure is negative.

Sample size	N/A
Data exclusions	No data were excluded from the analysis
Replication	At least three independent biological replicates were performed for all experiments (two or three technical replicates per experiment).
Randomization	N/A
Blinding	No blinding was performed as the experiments were performed and analyzed by the same person.

Reporting for specific materials, systems and methods

We require information from authors about some types of materials, experimental systems and methods used in many studies. Here, indicate whether each material, system or method listed is relevant to your study. If you are not sure if a list item applies to your research, read the appropriate section before selecting a response.

Materials & experimental systems

n/a	Involved in the study	
☐	☒	Antibodies
☐	☒	Eukaryotic cell lines
☒	☐	Palaeontology and archaeology
☒	☐	Animals and other organisms
☒	☐	Clinical data
☒	☐	Dual use research of concern
☒	☐	Plants

Methods

n/a	Involved in the study	
☒	☐	ChIP-seq
☒	☐	Flow cytometry
☒	☐	MRI-based neuroimaging

Antibodies

Antibodies used

PA (Da Costa et al., 2015 PMID: 25855727)
A rabbit serum directed against the PA domain (residues 197 to 257) was used to reveal PA expression. Validation: transfected with pCI-PA/pCI-empty HEK293T cells.
validation data included in the manuscript: control uninfected cells

PB2 (GTX125925, GeneTex)
https://www.genetex.com/Product/Detail/Influenza-A-virus-PB2-protein-antibody/GTX125925
validation data included in the manuscript: control uninfected cells

Histone H3: (#9715, Cell Signaling Technology)
https://www.cellsignal.com/products/primary-antibodies/histone-h3-antibody/9715
validation data shown on the provider's website:

Gaussia luciferase (#E8023, New England Biolabs)
https://www.neb.com/en/-/media/catalog/msds/s/d/s/e/8/sdse8023gh.pdf?rev=0e3e0b35f08c4852852fee4ce6d621d4&hash=44A813ACC9E74C8342CE774D267ACE57
validation data included in the manuscript: control untransfected cell

HRP-tagged secondary antibodies (Jackson Immunoresearch)

Validation

Validation of each primary antibody is described above

Eukaryotic cell lines

Policy information about cell lines and Sex and Gender in Research

Cell line source(s)

The 293T cells were purchased at ATCC (CRL-3216)
The 293T CTRL and ANP32AB KO cells were derived from 293T/17 cells purchased at ATCC (CRL-11268).
Trichoplusia ni High5 cells

Authentication

293T and 293T/17 cells were authentified by ATCC
Trichoplusia ni High5 cells (Commerically available cell line from ThermoFisher)

Mycoplasma contamination

Each cell line used has been tested on a regular basis for the absence of mycoplasma, using a specific PCR detection protocol.
Trichoplusia ni High5 cells (Not tested for mycoplasma)

Commonly misidentified lines
(See ICLAC register)

Name any commonly misidentified cell lines used in the study and provide a rationale for their use.

Plants

Seed stocks	N/A
Novel plant genotypes	N/A
Authentication	N/A
